# Hypothermic oxygenated perfusion inhibits CLIP1-mediated TIRAP ubiquitination via TFPI2 to reduce ischemia‒reperfusion injury of the fatty liver

**DOI:** 10.1038/s12276-024-01350-8

**Published:** 2024-12-02

**Authors:** Pengpeng Yue, Xiaoyan Lv, Hankun Cao, Yongkang Zou, Jian You, Jun Luo, Zhongshan Lu, Hao Chen, Zhongzhong Liu, Zibiao Zhong, Yan Xiong, Xiaoli Fan, Qifa Ye

**Affiliations:** 1 Zhongnan Hospital of Wuhan University, Institute of Hepatobiliary Diseases of Wuhan University, Transplant Center of Wuhan University, National Quality Control Center for Donated Organ Procurement, Hubei Key Laboratory of Medical Technology on Transplantation, Hubei Clinical Research Center for Natural Polymer Biological Liver, Hubei Engineering Center of Natural Polymer-based Medical Materials, 430071 Wuhan, China; 2 Department of Hematology, Zhongnan Hospital of Wuhan University, 430071 Wuhan, China; 3 Department of Hepatobiliary Surgery, Department of Organ Transplantation, Guizhou Provincial People’s Hospital, 550002 Guiyang, China; 4 The Third Xiangya Hospital of Central South University, Research Center of National Health Ministry on Transplantation Medicine Engineering and Technology, 410013 Changsha, China

**Keywords:** Ubiquitylation, Non-alcoholic fatty liver disease, Experimental models of disease, Chronic inflammation, Mechanisms of disease

## Abstract

The use of fatty livers in liver transplantation has emerged as a crucial strategy to expand the pool of donor livers; however, fatty livers are more sensitive to ischemia‒reperfusion injury (IRI). Excessive congenital inflammatory responses are crucial in IRI. Hypothermic oxygenated perfusion (HOPE) is a novel organ preservation technique that may improve marginal donor liver quality by reducing the inflammatory response. Tissue factor pathway inhibitor-2 (TFPI2) and CAP-Gly domain-containing linker protein 1 (CLIP1) exhibit modulatory effects on the inflammatory response. However, the underlying mechanisms of HOPE in fatty liver and the effects of TFPI2 and CLIP1 in fatty liver IRI remain unclear. Here, we aimed to explore the impact of HOPE on the inflammatory response in a rat model of fatty liver IRI and the mechanisms of action of TFPI2 and CLIP1. HOPE significantly reduces liver injury, especially the inflammatory response, and alleviates damage to hepatocytes and endothelial cells. Mechanistically, HOPE exerts its effects by inhibiting TFPI2, and CLIP1 can rescue the damaging effects of TFPI2. Moreover, HOPE promoted the ubiquitination and subsequent degradation of Toll/interleukin-1 receptor domain-containing adapter protein (TIRAP) by regulating the binding of R24 of the KD1 domain of TFPI2 with CLIP1, thereby negatively regulating the TLR4/NF-κB-mediated inflammatory response and reducing IRI. Furthermore, TFPI2 expression increased and CLIP1 expression decreased following cold ischemia in human fatty livers. Overall, our results suggest that targeting the inflammatory response by modulating the TFPI2/CLIP1/TIRAP signaling pathway via HOPE represents a potential therapeutic approach to ameliorate IRI during fatty liver transplantation.

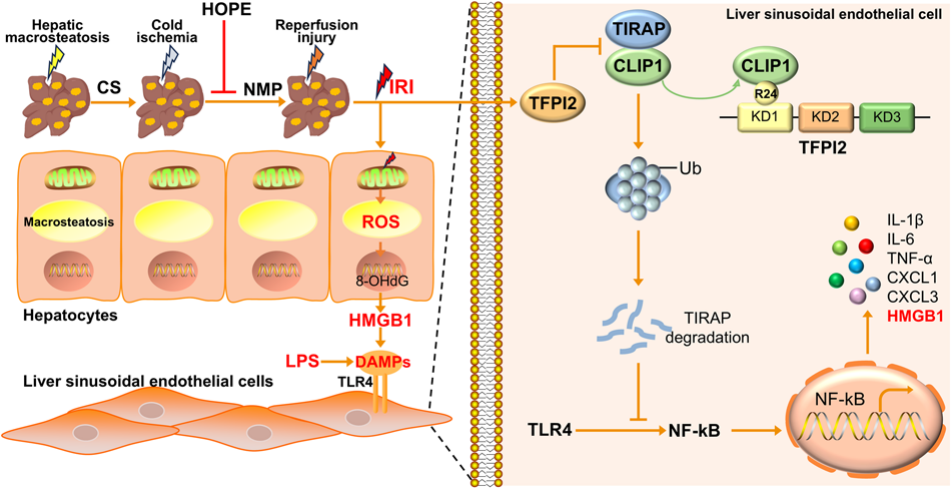

## Targeting Inflammation Enhances Fatty Liver Transplant Outcomes

Liver transplantation is the only effective treatment for end-stage liver disease, but donor organ shortages limit its progress. Researchers found that fatty livers, which make up 15-30% of donations, are more prone to damage during transplantation. In this study, the researchers used a rat model to explore how hypothermic oxygenated perfusion (HOPE) affects fatty liver transplants. HOPE is a technique where the liver is preserved at low temperatures with oxygenated fluid. They divided rats into three groups: sham, cold storage (CS), and HOPE. Results showed that HOPE significantly reduces liver injury, especially the inflammatory response, and alleviates oxidative stress to improve liver function. The researchers concluded that HOPE can protect fatty livers during transplantation by inhibiting inflammation. Future studies could explore HOPE’s potential in human liver transplants. This summary was initially drafted using artificial intelligence, then revised and fact-checked by the author.

## Introduction

Liver transplantation is the sole efficacious therapy for terminal liver disease. Nevertheless, the insufficiency of donor organs constrains the advancement of liver transplantation technologies^1^. The quality of donor livers has become a focus of attention in the field of organ transplantation because of the increasing number of organ donations each year. Among the donated organs, fatty liver is a substantial concern for poor liver quality, with an incidence rate of 15–30%^2^. The accumulation of fatty acids and neutral fats in liver cells leads to a specific change in the liver parenchyma known as steatosis. Steatosis is associated with liver damage caused by proapoptotic activity and inflammatory stimulation by free fatty acids^3^. Fatty livers are more susceptible to cold ischemia‒reperfusion injury (IRI) than normal livers. First, the liver cell volume increases, inhibiting the liver sinusoids during liver cell steatosis, causing a relatively ischemic environment within the fatty liver. Second, insufficient mitochondrial beta-oxidation in fatty livers reduces ATP generation in liver cells, thereby decreasing energy in the liver during IRI and increasing reactive oxygen species (ROS) production. Third, abnormal regulation of Kupffer cells in fatty livers decreases the regulation of liver sinusoidal blood flow^4,5^.

During IRI, endothelial cells (ECs) are more susceptible to damage because of the leakage of damage-associated molecular patterns (DAMPs), such as endogenous DNA or high mobility group box 1 (HMGB1), from damaged steatotic hepatocytes, which activate the TLR inflammatory pathway in ECs, leading to the release of inflammatory cytokines^6,7^. Extracellular vesicles secreted by steatotic hepatocytes contain proteins, lipids, and nucleic acids that further promote EC inflammation and injury^8^.

These factors contribute to a 35–40% increase in the risk of severe complications [such as delayed graft function (DGF) and primary nonfunction (PNF)] and liver function failure after liver transplantation^9^. Macrosteatosis exceeding 60% is a contraindication for liver transplantation. However, in clinical practice, postoperative PNF significantly increases to 13% when macrosteatosis exceeds 30%, with a mortality rate of up to 14%^10,11^.

Excessive innate inflammatory responses have attracted particular attention owing to the significant molecular events involved in IRI. DAMPs released by stressed cells bind to pattern recognition receptors, activating natural immune cells^12,13^. Toll-like receptor 4 (TLR4) is essential in sterile inflammation^13^. Inhibition of the TLR4 signaling pathway can alleviate IRI in donations after circulatory death (DCD) in rat livers^14^. In fatty liver transplantation, reperfusion injury activates the TLR4 signaling pathway, leading to downstream inflammatory responses^6^. The Toll/interleukin-1 receptor domain-containing adapter protein (TIRAP) is a signaling molecule that regulates various immune responses and acts as an adapter protein that couples myeloid differentiation factor 88 (MyD88) with TLRs, activating the TLR4/MyD8/NF-κB signaling pathways, thereby leading to proinflammatory responses^15^. Additionally, TIRAP is involved in several inflammation-related protein‒protein interactions^16^. For example, when the receptor for advanced glycation end products binds to TIRAP, it activates the NF-kB and AP-1 inflammatory pathways^17^; P38 MAPK and TIRAP interactions activate AP-1^18^; TIRAP and PI3K interactions activate the PI3K‒Akt pathway^19^; and CLIP170 and TIRAP interactions reduce NF-κB and AP-1 responses^20^. The involvement of TIRAP in several intracellular signaling pathways indicates its important role in various inflammatory responses, making it a potential therapeutic target for inhibiting acute and chronic inflammation.

Hypothermic oxygenated perfusion (HOPE) is a novel organ preservation technique for donor livers and a promising method to protect against marginal liver IRI^21^. HOPE alleviates DCD liver IRI by regulating inflammation, oxidative stress, and other factors^22–24^. HOPE treatment during IRI in the fatty liver significantly reduces posttransplantation IRI, which is characterized by decreased oxidative stress, nuclear damage, necrosis, EC activation, and fibrosis^6,25^.

Damage from prolonged cold IRI causes ROS accumulation in the fatty liver, TLR signaling activation, and worsening inflammation^6^. Although HOPE mitigates fatty liver injury and suppresses the inflammatory response, the specific molecular mechanisms involved remain unclear. Therefore, this study aimed to establish a rat model of fatty liver disease and explore the effect of HOPE on TLR4-mediated inflammatory responses during fatty liver IRI and its mechanism of action, thereby providing new intervention targets for treating IRI during fatty liver transplantation.

## Materials and methods

### Animals

SD rats (adult, male, 300–350 g) were provided by Wanqian Jiaxing Biotechnology Co., Ltd., Wuhan, China. The rats were housed in a pathogen-free environment at the Animal Laboratory Center, Zhongnan Hospital of Wuhan University. They were fed a methionine‒choline-deficient (MCD) diet (10% fat content, purchased from Xietong Pharmaceutical Bioengineering Co., Ltd., Jiangsu, China.) for 2 weeks to establish a fatty liver model (≥60% macrosteatosis). The animals had unrestricted access to water and food during the entire research period.

This study received approval from the Experimental Animal Welfare Ethics Committee at Zhongnan Hospital of Wuhan University (approval no. ZN2022202). Animal experiments were carried out in strict adherence to the Regulations on Laboratory Animal Management (issued by the National Science and Technology Commission of China) and the Guidelines for Laboratory Animal Care and Use (National Institutes of Health in Bethesda, MD, USA).

### Study design

Sodium pentobarbital (30 mg/kg) was injected intraperitoneally into the rats to anesthetize them. Their skin was prepared, disinfected, and draped. The skin, subcutaneous tissues, muscles, and peritoneum were sequentially incised along the midline of the abdomen to expose the liver. A 100 mL 4 °C histidine–tryptophan–ketoglutarate (HTK) solution was slowly perfused through the abdominal aorta, and the liver was cooled with 4 °C saline. After the liver was thoroughly washed, it was isolated intact, the portal vein and infrahepatic inferior vena cava were cannulated, the suprahepatic inferior vena cava was ligated, and the livers were preserved in HTK solution at 4 °C.

**Fig. 1:**
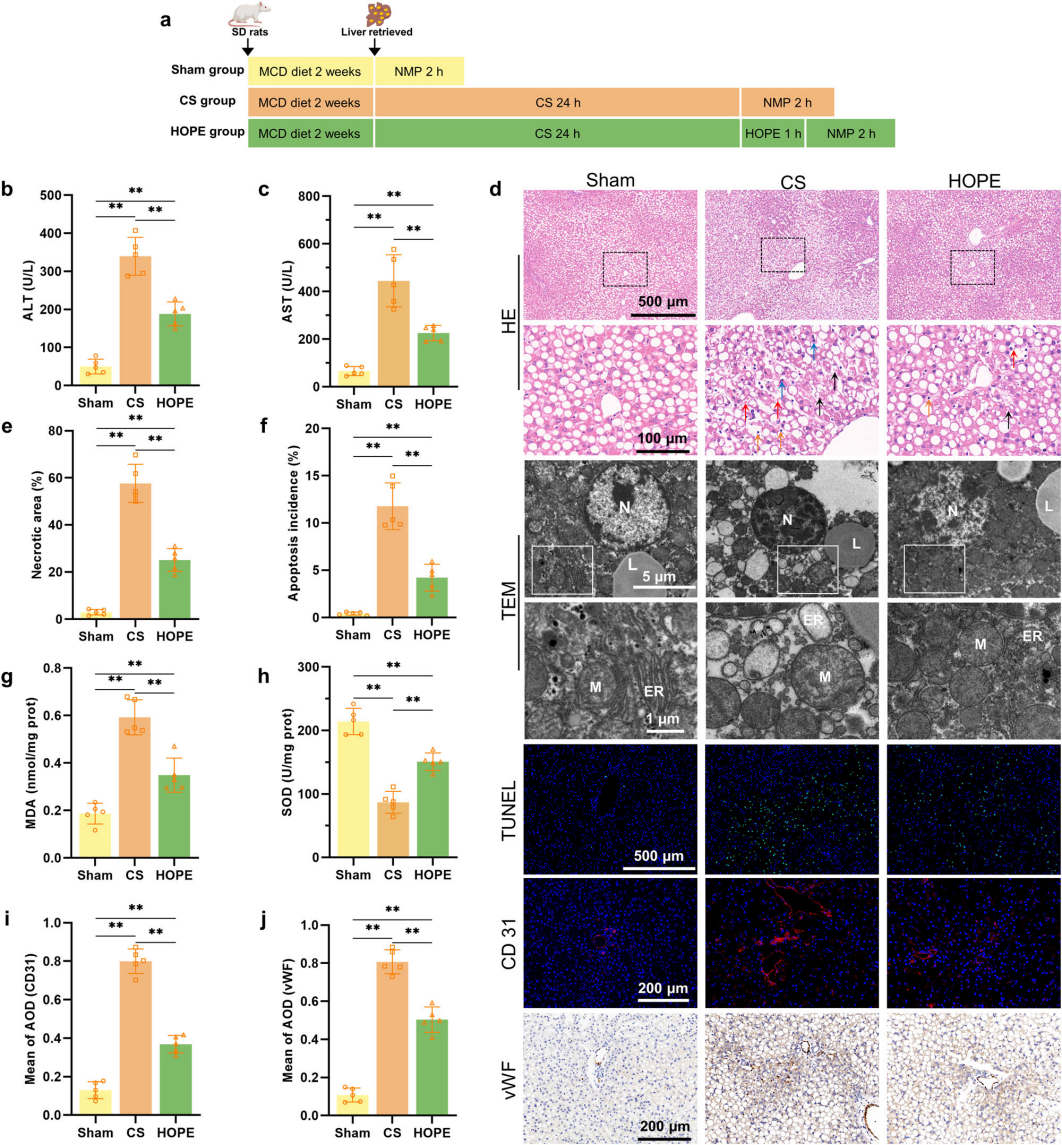
HOPE improves fatty liver function and alleviates tissue damage. **a** Experimental design. **b**, **c** Concentrations of ALT and AST in the perfusate. **d** Liver tissue HE staining, TEM images, TUNEL and IF staining for CD31, and IHC for vWF. The black arrows indicate cavitation, the red arrows indicate red blood cells, the blue arrows indicate neutrophils, and the orange arrows indicate nuclear shrinkage. N represents the nucleus, M represents the mitochondria, ER represents the endoplasmic reticulum, and L represents lipid droplets. **e** Necrotic area analysis of liver histology. **f** Apoptotic ratio analysis of TUNEL-stained cells. **g**, **h** Concentrations of MDA and SOD in liver tissue. **i** Results from the quantitative analysis of CD31 via IF. **j** Quantitative analysis of vWF via IHC. *n* = 5 per group; the data are presented as the means ± SDs; ns not significant; *P < 0.05; and **P < 0.01.

The rats were divided into three groups as follows: the sham, cold storage (CS), and HOPE groups. Immediately after fatty liver was induced, the sham group underwent 2 h of normothermic reperfusion (NMP); the CS group underwent 24 h of CS in HTK solution at 4 °C, followed by 2 h in NMP; and the HOPE group underwent 24 h of CS, 1 h of HOPE, and 2 h of NMP (Fig. 1a).

The HOPE and NMP systems were modified on the basis of previous descriptions^23,24^. In general, the HOPE device consists of a peristaltic pump, an oxygenator, a water bath, pressure sensors, and perfusion tubing. The HTK solution is used as the perfusion fluid, flowing into the portal vein and exiting through the inferior vena cava. The temperature is maintained at 0–4 °C, with a portal vein perfusion pressure of 2–3 mmHg, a perfusion fluid flow rate of 0.23 mL/min/g, and oxygen maintained at approximately 200 mmHg. The duration of HOPE was 1 h. The NMP system is similar to that of HOPE, except that the perfusion fluid is Krebs‒Henseleit buffer (KHB) solution, with the temperature controlled at 35‒37 °C, portal vein pressure at 8‒10 mmHg, perfusion fluid flow rate of 2 mL/min/g, and a gas mixture of 95% oxygen and 5% carbon dioxide, which is maintained at ~500 mmHg. The duration of NMP was 2 h.

### Preparation of AAV8

TFPI2-overexpressing (ov-TFPI2), TFPI2-knockdown (sh-TFPI2), and empty vector (NC) of adeno-associated virus serotype 8 (AAV8) were provided by OBiO Technology Co., Ltd. (Shanghai, China). AAV8 (5 × 10^11^ v/mL per rat) was administered via tail vein injection.

### Cell culture

Human umbilical vein endothelial cells (HUVECs), buffalo rat liver-3A cells (BRL-3A), and human embryonic kidney 293T (HEK293T) cells were cultured at 37 °C in a 5% CO_2_ environment in Dulbecco’s modified Eagle’s high-glucose medium supplemented with 1% penicillin‒streptomycin and 10% fetal bovine serum. In accordance with the experimental design, lipopolysaccharide (LPS) (Sigma, USA) was applied to the cells for 24 h.

### Vector construction, cell transfection, and lentiviral infection

The plasmids encoding TFPI2, His-TFPI2, His-TFPI2(ΔKD1), His-TFPI2(ΔKD2), His-TFPI2(ΔKD3), His-TFPI2(R24Q), Flag-CLIP1, Myc-TIRAP, and HA-Ub were constructed by Miaoling Biotechnology Co., Ltd., Wuhan, China. Cell transfection was performed using NEOFECT DNA transfection reagent (Beijing, China).

To establish a stable TFPI2-knockdown cell line, a shRNA lentivirus was prepared by cotransfecting HEK293T cells with GL427 and the packaging plasmids pMD2G and psPAX2 (OBiO, Shanghai, China). After 48 h of transfection, the viral supernatant was collected. HUVECs were incubated with the viral supernatant in the presence of 8 μg/mL polybrene. Positive cells were selected with 2.5 μg/mL puromycin. The sequence of shTFPI2 is listed in Supplementary Table 1.

### Aspartate aminotransferase (AST), alanine aminotransferase (ALT), malondialdehyde (MDA), and superoxide dismutase (SOD) detection

ALT and AST in the perfusate samples of each group were detected by an automatic biochemical analyzer (Chemray 80, Guangdong, China). The levels of MDA and SOD in fresh liver tissue samples from each group were measured using reagent kits (Jiancheng, Nanjing, China) according to the manufacturer’s instructions.

### Hematoxylin and eosin (HE), Oil red O (ORO), terminal deoxynucleotidyl transferase dUTP nick-end labeling (TUNEL), immunohistochemistry (IHC), and immunofluorescence (IF) staining

Fresh liver tissue was preserved in paraformaldehyde (4%) for 24 h, encased in paraffin, and then sliced into sections. The sections were stained with HE or ORO staining solutions for microscopic observation. Hepatocyte apoptosis was measured using TUNEL staining. IHC was performed by sequentially incubating paraffin sections with specific antibodies, staining them with 3,3′-diaminobenzidine (DAB), counterstaining them with hematoxylin, and examining them under a microscope. For IF, cells cultured on coverslips or paraffin sections fixed with paraformaldehyde were incubated with specific primary and fluorescent secondary antibodies and observed under a fluorescence microscope after counterstaining with 4’,6-diamidino-2-phenylindole. The primary antibodies used for IHC and IF were as follows: anti-vWF (GB11020), anti-MCP1 (GB11199), anti-CD31 (GB120633), anti-CD206 (GB11527), anti-MPO (GB11224), and anti-CD11b (GB15058) provided by Service Biotechnology Co., Ltd., Wuhan, China; the anti-TFPI2, anti-CLIP1, anti-TIRAP, anti-TLR4, and anti-HMGB1 antibodies were the same as those used for Western blotting.

### Transmission electron microscopy (TEM)

Fresh liver tissue (1–2 mm^2^) was preserved in an electron microscope-fixing liquid (Servicebio, Wuhan, China) for 2 h. After gradient dehydration with alcohol, acetone infiltration, epoxy resin embedding, slicing, and staining, the specimens were observed and photographed using a Hitachi HT7700 TEM (Japan) at 80 kV.

### Enzyme-linked immunosorbent assay (ELISA)

The ELISA kits for IL-6, IL-10, IL-1β, HMGB1, and 8-OHdG were purchased from Service Biotechnology Co., Ltd., Wuhan, China. The supernatant of the liver tissue homogenate or cell culture medium was collected via centrifugation at 1200 rpm for 10 min at 4 °C. The assay was performed according to the manufacturer’s instructions. The OD of each well was detected at 450 nm.

### ROS assay

Intracellular ROS were detected via a ROS assay kit (Beyotime, Shanghai, China). DCFH-DA was diluted 1000 times with serum-free culture medium, added to the cells, and incubated for 20 min at 37 °C. After the cells were washed three times, they were observed and photographed with a fluorescence microscope (Nikon Eclipse C1, Japan).

### Flow cytometry (apoptosis) detection

The cells of each group were digested with trypsin without ethylenediaminetetraacetic acid. Each cell pellet was resuspended in 1× binding buffer per tube (100 μL), followed by the addition of V-fluorescein isothiocyanate (5 μL) and propidium iodide (5 μL) (Elabscience, E-CK-A211, Wuhan, China) in the dark. The sample was mixed thoroughly and incubated at 25 °C for 15 min. The sample was subsequently centrifuged, and the supernatant was collected. The pellet was washed with PBS once or twice, and the cells were resuspended in 300 μL of 1× binding buffer, mixed thoroughly, and analyzed using a flow cytometer (Cytolfex, Beckman Coulter, USA).

### Quantitative real‑time polymerase chain reaction (RT-qPCR) and RNA sequencing (RNA-seq)

Total RNA was extracted from the liver using TRIzol reagent (Invitrogen, USA). Using a one-step gDNA removal reverse transcription kit (Servicebio, Wuhan, China), the mRNA was converted to cDNA. RT-qPCR was performed using SYBR Green (Servicebio, Wuhan, China) to intercalate fluorescence. β-actin was utilized as the reference gene. The primers used for all target genes are listed in Supplementary Table 1.

The extracted RNA was sequenced on the HiSeq platform using the Illumina TruSeq RNA Sample Prep Kit for library construction. Genomic information on *Rattus norvegicus* was obtained from http://asia.ensembl.org/Rattus_norvegicus/Info/Index. Differential gene expression was calculated on the basis of gene read count data and analyzed using edgeR software. The selection criteria for significantly differentially expressed genes (DEGs) were a false discovery rate < 0.05 and |log_2_ fold change | ≥1.5.

### Western blotting

The western blotting experimental procedures were described previously^24^. The primary antibodies used in this study were as follows: anti-TFPI2 (1:4000, Abcam, ab186747, Cambridge, UK), anti-CLIP1 (1:1000, Proteintech, 23839-1-AP, Wuhan, China), anti-TIRAP (1:1000, Abcam, ab17218, Cambridge, UK), anti-IL-1β (1:5000, Proteintech, 16806-1-AP, Wuhan, China), anti-TNF-α (1:1000, Proteintech, 60291-1-Ig, Wuhan, China), anti-HMGB1 (1:3000, Proteintech, 10829-1-AP, Wuhan, China), anti-TLR4 (1:1000, Proteintech, 19811-1-AP, Wuhan, China), anti-p65 (1:3000, Proteintech, 10745-1-AP, Wuhan, China), anti-phospho-p65 (Ser536) (1:500, Affinity Biosciences, AF2006, Shanghai, China), anti-IκBα (1:5000, Proteintech, 10268-1-AP, Wuhan, China), anti-phospho-IκBα (Ser32/Ser36) (1:300, Affinity Biosciences, AF2002, Shanghai, China), anti-ubiquitin (1:300, Proteintech, 10201-2-AP, Wuhan, China), anti-His tag (1:5000, Proteintech, 66005-1-AP, Wuhan, China), anti-MYC tag (1:5000, Proteintech, 16286-1-AP, Wuhan, China), anti-HA tag (1:5000, Proteintech, 51064-2-AP, Wuhan, China), anti-Flag tag (1:20000, Proteintech, 20543-1-AP, Wuhan, China), anti-GST tag (1:2000, Proteintech, 10000-0-AP, Wuhan, China), and anti-β-actin (1:10,000, Abclonal, AC026, Wuhan, China).

### Coimmunoprecipitation (CoIP) and liquid chromatography‒tandem mass spectrometry (LC‒MS)

For CoIP, lysis buffer was added to the cells or liver tissue at 4 °C, the mixture was centrifuged at 12,000 rpm for 10 min, and the supernatant was collected. The corresponding primary antibody (10 μg) was added and incubated overnight at 4 °C to form antigen‒antibody complexes. The antigen‒antibody complexes were mixed with protein A/G MagPoly (ACE Biotechnology, Inc., Jiangsu, China) and incubated at 25 °C for 2 h. After washing, elution, and denaturation of the proteins bound to the beads, SDS-PAGE was performed. The gels were subjected to Western blotting and silver staining. Proteins in the samples were identified via LC‒MS. The gel was washed and dehydrated, trypsin was added, and the resulting mass spectrometry samples were analyzed using a mass spectrometer (Ultraflex III, Bruker, Germany). Data analysis was performed using MaxQuant (V2.2.0.0, Germany).

### Human liver tissue samples

Human liver samples were obtained from Wuhan University Zhongnan Hospital. Approval for the study was obtained from the Medical Ethics Committee of the hospital (approval no. 2020122), and informed consent was obtained from all participants.

### Statistical methods

Statistical analyses were performed using GraphPad Prism 8.0.2 software, and all of the data are presented as the mean ± standard deviation (mean ± SD). Student’s *t*-test was used for comparing two groups, whereas one-way or two-way analysis of variance (ANOVA) was used for comparing multiple groups, followed by Tukey’s post hoc test. A significance level of *p* < 0.05 was applied.

## Results

### HOPE improves fatty liver function and tissue damage

We established a steatosis model using BRL-3A cells to validate the high sensitivity of fatty liver to IRI. Compared with normal cells, steatotic cells presented further increases in the levels of inflammation (IL-1β, IL-6, TNF-α, and HMGB1) and ROS under CS conditions (Supplementary Fig. 1a–f). Ex vivo livers subjected to prolonged CS presented significantly higher levels of AST and ALT in the steatosis group than in the control group in the rat model of MCD-induced fatty liver (Supplementary Fig. 2a, b). HE staining revealed increased cell necrosis and inflammatory cell infiltration (Supplementary Fig. 2c–e). These results indicated that steatotic livers subjected to cold IRI presented more severe damage.

We measured liver enzyme levels in the perfusate to assess the role of HOPE in fatty liver IRI. Compared with the sham group, the CS group presented significant increases in AST and ALT levels, which were reduced after HOPE treatment (Fig. 1b, c). HE staining of liver tissues revealed significant pathological damage, including inflammatory cell infiltration, vacuolization, and hepatocyte necrosis, in the CS group, whereas the HOPE group presented significantly lower levels of pathological damage (Fig. 1d, e). ORO staining revealed no significant difference in the proportion of lipid droplets among the three groups (Supplementary Fig. 3), indicating that HOPE did not alleviate hepatic steatosis, which is consistent with the findings of previous studies^6^. The TUNEL staining results were consistent with the HE staining results. Hepatocyte apoptosis increased in the CS group but decreased in the HOPE group (Fig. 1d, f). Compared with the sham group, the CS group presented more severe nuclear condensation, mitochondrial swelling, disrupted mitochondrial cristae, mitochondrial matrix vacuolization, and endoplasmic reticulum expansion, according to TEM. This structural damage was significantly reduced in the HOPE group (Fig. 1d). IRI can induce significant oxidative stress in liver tissues^26^ owing to the significantly increased MDA levels and decreased SOD levels in the CS group. HOPE treatment markedly reduced hepatic oxidative stress levels in fatty livers (Fig. 1g, h).

We performed IF and IHC staining for CD31 and vWF, respectively, to explore the effects of HOPE on the homeostasis of liver sinusoidal endothelial cells (LSECs). The levels of CD31 and vWF were increased in the CS group, whereas the increase in the HOPE group was significantly inhibited compared with those in the sham group (Fig. 1d, i, j). These findings indicate that HOPE can alleviate IRI-induced damage to LSECs.

These findings suggested that HOPE significantly improved fatty liver function, alleviated liver tissue damage, inhibited oxidative stress, and protected LSECs.

### HOPE reduces inflammation levels in the fatty liver

**Fig. 2:**
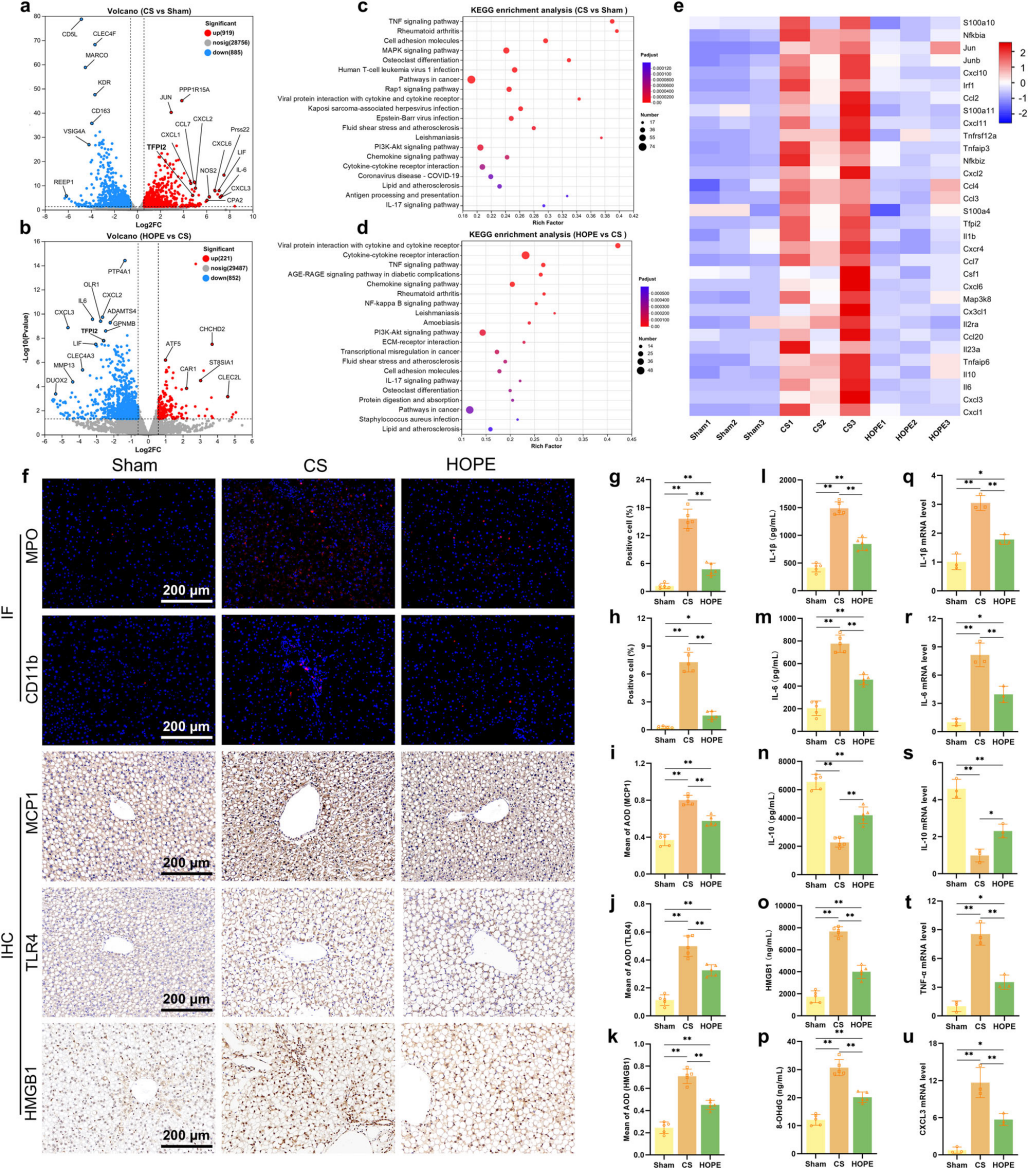
HOPE reduces inflammation in the fatty liver. **a**, **b** Volcano map of liver DEGs (*n* = 3 per group). **c, d** KEGG pathway enrichment analysis of liver DEGs (*n* = 3 per group). **e** Heatmap analysis of genes associated with inflammation (*n* = 3 per group). **f** IF staining of MPO and CD11b and IHC staining of MCP1, TFLR4, and HMGB1 in liver tissue (*n* = 5 per group). **g**, **h** Proportions of MPO and CD11b IF-positive cells (*n* = 5 per group). **i–k** Quantitative IHC analysis of MCP1, TFLR4, and HMGB1 (*n* = 5 per group). **l‒p** Concentrations of IL-1β, IL-6, IL-10, HMGB1, and 8-OHdG in liver tissue were determined via ELISA (*n* = 5 per group). **q–u** RT‒qPCR analysis of IL-1β, IL-6, IL-10, TNF-α, and CXCL3 mRNA levels in liver tissue (*n* = 3 per group). Data are presented as the mean ± SD; ns not significant; *P < 0.05; and **P < 0.01.

We collected rat liver samples from the sham, CS, and HOPE groups for RNA-seq analysis to further investigate the role of HOPE in fatty liver IRI. A comparative analysis between the CS and sham groups revealed 919 upregulated and 885 downregulated genes. A total of 221 genes were upregulated, and 852 genes were downregulated when the HOPE group was compared with the CS group (Fig. 2a, b; Supplementary Fig. 4a). Gene Ontology (GO) enrichment analysis revealed that the DEGs were enriched in pathways involved in monocyte chemotaxis, leukocyte migration, leukocyte chemotaxis, neutrophil migration, and leukocyte apoptotic processes (Supplementary Fig. 4b, c). Kyoto Encyclopedia of Genes and Genomes (KEGG) enrichment analysis revealed that the DEGs were enriched in the TNF, IL-17, NF-κB, MAPK, chemokine, and PI3K-Akt pathways (Fig. 2c, d). Furthermore, a heatmap revealed significantly greater expression of inflammation-related genes in the liver tissue of the CS group than in that of the sham group, with downregulation of these genes upon HOPE treatment (Fig. 2e). These findings suggest that HOPE may regulate the inflammatory response to improve fatty liver IRI. IF staining of the neutrophil markers CD11b and MPO revealed a significant increase in neutrophil infiltration in the CS group, which was partially reversed by HOPE in fatty liver subjected to IRI (Fig. 2f–h). HOPE treatment significantly reduced the accumulation of MCP1 induced by fatty liver IRI as shown by IHC (Fig. 2f, i). The upregulation of TLR4 and HMGB1 expression in the CS group was decreased by HOPE treatment according to the IHC results (Fig. 2f, j, k). Compared with those in the CS group, the levels of IL-1β, IL-6, HMGB1, and 8-OHdG were significantly lower in the HOPE group, whereas the IL-10 levels were greater (Fig. 2l–p). Moreover, the mRNA levels of TNF-α, IL-1β, IL-2, IL-6, CXCL1, and CXCL3 were lower in the HOPE group than in the CS group, with IL-4 and IL-10 showing the opposite trend (Fig. 2q–u, Supplementary Fig. 5). These data suggest that HOPE reduces fatty liver IRI in rats by inhibiting the inflammatory response, with a significant inhibitory effect on TLR4-mediated inflammation.

### HOPE improved fatty liver function and damage by inhibiting TFPI2

**Fig. 3:**
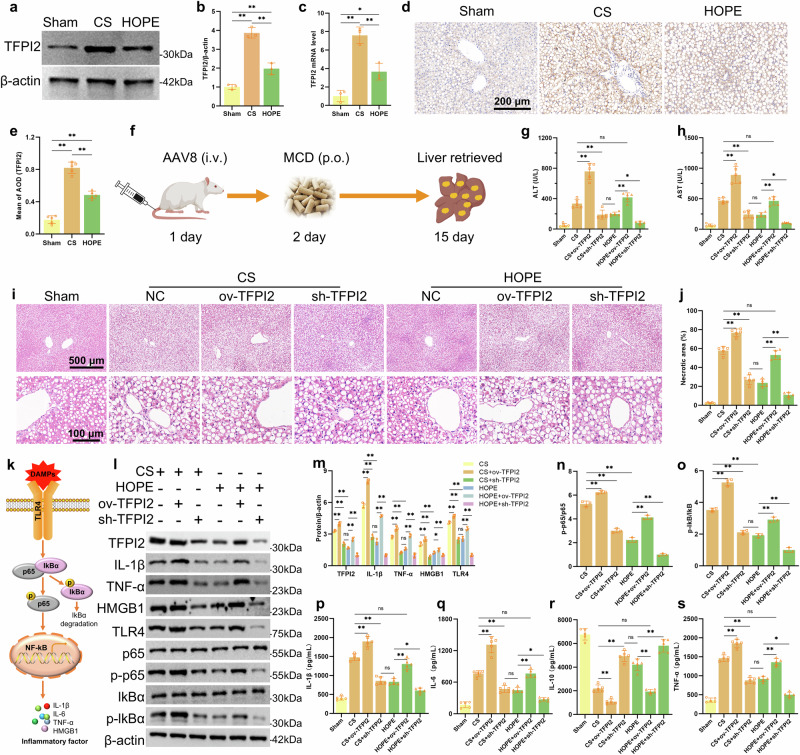
HOPE improves fatty liver function and damage by inhibiting TFPI2. **a**, **b** Western blotting and statistical analyses of the TFPI2 protein in liver tissue (*n* = 3 per group). **c** RT‒qPCR was used to detect TFPI2 mRNA levels in liver tissue (*n* = 3 per group). **d**, **e** IHC staining and statistical analysis of TFPI2 in liver tissue (*n* = 5 per group). **f** Diagram of the SD rat fatty liver model and AAV8 injection. ov-TFPI2, sh-TFPI2, or empty vector AAV8 was injected into SD rats through the tail vein; an MCD diet was started on the second day after the AAV8 injection, and the liver was retrieved on the 15th day for the following experiments. **g**, **h** Concentrations of ALT and AST in the perfusate (*n* = 5 per group). **i**, **j** HE staining and histological necrotic area analysis of liver tissue (*n* = 5 per group). **k** Schematic diagram of the TLR4 and NF-κB inflammatory signaling pathways. **i‒o** Western blotting and statistical analyses of TFPI2, IL-1β, TNF-α, HMGB1, TLR4, p65, p-p65, lkB, p-lkB, and β-actin proteins in liver tissue (*n* = 3 per group). **p–s** IL-1β, IL-6, IL-10, and TNF-α concentrations in liver tissue as determined by ELISA (*n* = 5 per group). The data are presented as the mean ± SD; ns not significant; *P < 0.05; and **P < 0.01.

DEG analysis revealed significant changes in the expression of the tissue factor pathway inhibitor-2 (TFPI2) gene, which was upregulated in the CS group and downregulated in the HOPE group (Fig. 2a, b). This gene is a member of the Kunitz-type serine protease inhibitor family, is synthesized primarily by ECs of small blood vessels, and is widely expressed in the liver, skeletal muscle, heart, kidneys, and pancreas. It induces apoptosis, participates in inflammatory responses, and inhibits angiogenesis^27–31^. The role of TFPI2, especially in inflammatory responses, involves its participation in LPS-induced liver inflammation in mice^27^. Extracellular stimuli, such as IL-1, TNF-α, sorbitol, anisomycin, and UV light, can activate the JNK, NF-κB, and p38 MAPK pathways through TFPI2^32^. These findings strongly suggest that TFPI2 is involved in the regulation of inflammatory responses. We observed that the TFPI2 expression level in the liver tissue of the CS group was greater than that in the sham group, and HOPE treatment attenuated this trend (Fig. 3a, b). The mRNA expression level of this gene showed the same trend (Fig. 3c). HOPE reduced the expression of TFPI2 as shown by IHC (Fig. 3d, e).

We transfected AAV8 constructs overexpressing or knocking down TFPI2 expression in rats fed an MCD diet to analyze the role of TFPI2 during fatty liver IRI (Fig. 3f). AAV8 showed the highest transfection efficiency 15 days after tail vein injection, with no effect on liver function (Supplementary Fig. 6). ov-TFPI2 AAV8 significantly increased AST and ALT levels in the CS and HOPE groups, whereas sh-TFPI2 AAV8 decreased AST and ALT levels in all groups (Fig. 3g, h). TFPI2 overexpression exacerbated pathological injuries, such as inflammatory cell infiltration, hepatocyte necrosis, and vacuolization in liver tissue, whereas silencing TFPI2 had the opposite effect according to HE staining (Fig. 3i, j).

We initially examined the levels of TLR4 and NF-κB inflammatory signaling-related proteins in liver tissue to investigate the role of TFPI2 in the regulation of inflammatory responses during fatty liver IRI (Fig. 3k). TFPI2 overexpression significantly increased the protein expression of TNF-α, IL-1β, HMGB1, TLR4, p-P65, and p-IκBα in the CS and HOPE groups, whereas TFPI2 silencing resulted in the opposite trend (Fig. 3l–o). Significantly increased levels of the proinflammatory cytokines TNF-α, IL-1β, and IL-6 in liver tissue were observed following ov-TFPI2 AAV8 administration, whereas significantly decreased levels of the anti-inflammatory cytokine IL-10 were detected via ELISA. These effects were reversed by sh-TFPI2 AAV8 treatment (Fig. 3p–s). HOPE alleviated the inflammatory response in fatty liver IRI by inhibiting TFPI2 expression, which mainly involves regulating the TLR4/NF-κB inflammatory signaling pathways.

### HOPE regulates the interaction between TFPI2 and CLIP1

**Fig. 4:**
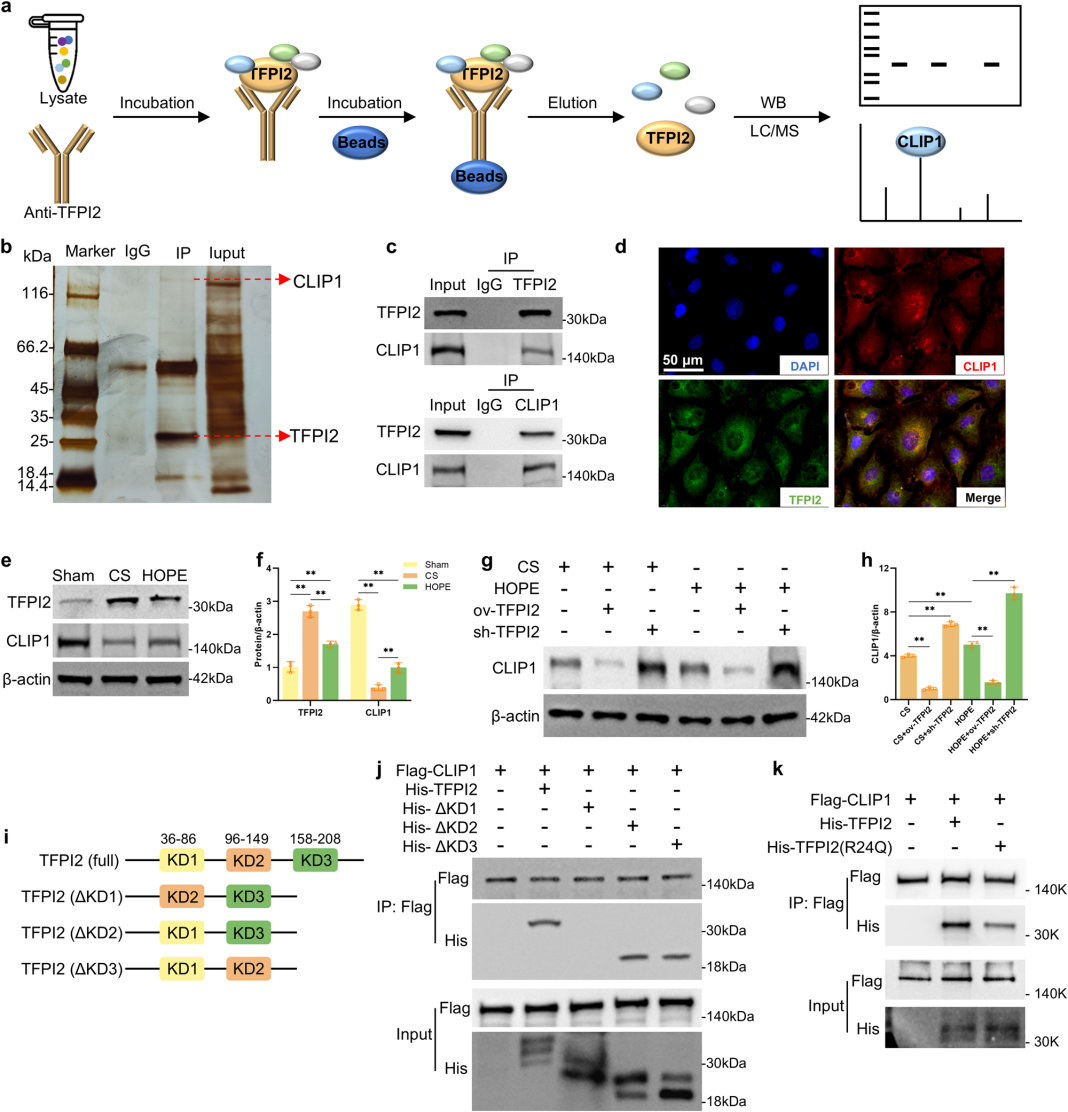
HOPE regulates the interaction between TFPI2 and CLIP1. **a** Schematic diagram of the identification of proteins that interact with TFPI2 via CoIP combined with LC‒MS. **b** Liver tissue lysate was incubated with an anti-IgG antibody (IgG) or anti-TFPI2 antibody (IP). Different bands of anti-TFPI2 and anti-IgG were detected in silver-stained SDS‒PAGE gels. **c** The interaction between TFPI2 and CLIP1 was confirmed by CoIP. **d** IF analysis of TFPI2 and CLIP1 in HUVECs (*n* = 5 per group). **e**, **f** Western blotting and statistical analyses of the TFPI2 and CLIP1 proteins in liver tissue (*n* = 3 per group). **g**, **h** Western blotting and statistical analyses of the CLIP1 protein in the liver tissue of rats infected with ov-TFPI2, sh-TFPI2, or empty vector AAV8 (n = 3 per group). **i** Schematic diagram of the full-length and deleted domain architecture of TFPI2. The KD1 domain is located in amino acid (aa) sequences 36 to 86, the KD2 domain is located in aa sequences 96 to 149, and the KD3 domain is located in the sequence 158 to 208. Δ means deleted. **j**, **k** CoIP analysis of the domains and sites where CLIP1 interacts with TFPI2. Flag-tagged CLIP1, His-tagged TFPI2, KD1, KD2, KD3, and mutant TFPI2 (R24Q) plasmids were transfected into HEK293T cells. The cell protein extract was tested via CoIP with an anti-FLAG primary antibody. The data are presented as the mean ± SD; ns not significant; *P < 0.05; and **P < 0.01.

We used CoIP combined with LC‒MS technology to identify the proteins that interact with TFPI2 to investigate the potential molecular mechanisms by which TFPI2 regulates inflammatory responses (Fig. 4a). CoIP samples on silver-stained SDS‒PAGE gels presented specific differential bands for IP (TFPI2) compared with those of the IgG control (Fig. 4b). LC‒MS analysis of the gel slices revealed 126 differentially expressed proteins. Among the candidate proteins listed in Supplementary Table 2, CAP-Gly domain-containing linker protein 1 (CLIP1) had the highest score and is known to negatively regulate TLR4-mediated inflammatory responses^20^. Hence, it was selected for further analysis. CLIP1 (also known as CLIP170) is a microtubule-associated protein containing two conserved CAP-Gly domains rich in glycine and two tandem repeat zinc finger motifs. CLIP1 is a multifunctional protein that specifically binds to and regulates the dynamic growth of microtubules plus ends^33–35^. We confirmed the interaction between TFPI2 and CLIP1 via CoIP (Fig. 4c), and IF staining revealed their colocalization in HUVECs (Fig. 4d). CLIP1 expression decreased in the CS group but increased after HOPE treatment compared with that in the sham group, which was opposite to the expression trend of TFPI2 (Fig. 4e, f). TFPI2 overexpression led to decreased CLIP1 protein expression when TFPI2 was silenced, resulting in increased CLIP1 expression in the CS and HOPE groups (Fig. 4g, h).

We constructed an exogenous recombinant expression system for CLIP1 and TFPI2, as well as mutant TFPI2 lacking functional domains (KD1: 36–86 aa, KD2: 96–149 aa, KD3: 158–208 aa), to further investigate the interaction between TFPI2 and CLIP1 (Fig. 4i). Flag-CLIP1 could not interact with TFPI2 lacking the KD1 domain, whereas the deletion of the KD2 and KD3 domains did not affect their binding, according to CoIP experiments (Fig. 4j). These findings indicate that the KD1 domain of TFPI2 directly interacts with CLIP1. The arginine at position 24 (R24) of the KD1 domain is considered the main active site of TFPI2^30^. After cells were transfected with the TFPI2 (R24Q) mutant plasmid, CoIP analysis of cellular proteins revealed a significant decrease in the binding of TFPI2 to CLIP1 (Fig. 4k). These data suggested that TFPI2 primarily interacted with CLIP1 at the R24 site of its KD1 domain.

### CLIP1 rescues the damaging effects of TFPI2

**Fig. 5:**
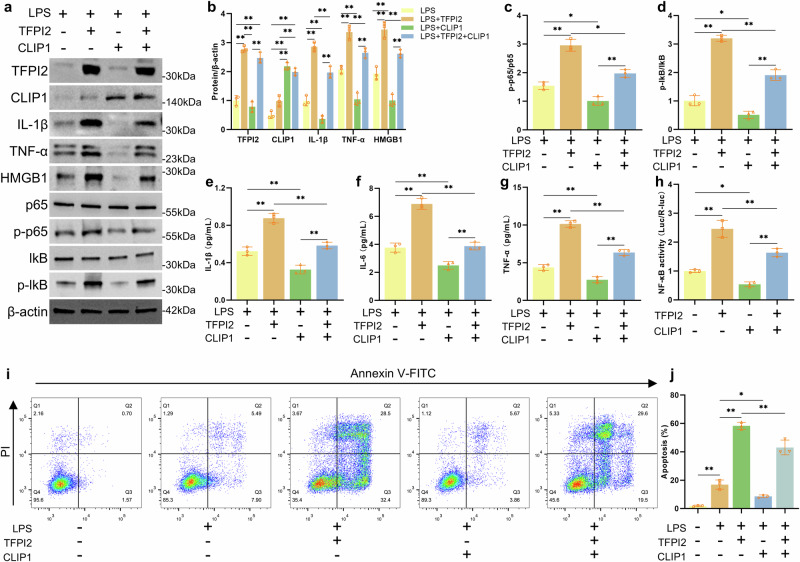
CLIP1 rescues the damaging effects of TFPI2. **a‒d** Western blotting and analysis of TFPI2, CLIP1, IL-1β, TNF-α, HMGB1, p65, p-p65, lkB, p-lkB, and β-actin proteins in HUVECs treated with LPS and transfected with TFPI2, CLIP1, or empty vector. **e–g** The concentrations of IL-1β, IL-6, and TNF-α in the culture medium of HUVECs treated with LPS and transfected with TFPI2, CLIP1, or empty vector were determined via ELISA. **h** NF-κB-responsive reporter activity in HEK293T cells transfected with TFPI2 and CLIP1. **i** and **j** Apoptosis of HUVECs treated with LPS and transfected with TFPI2, CLIP1, or empty vector detected by flow cytometry. *n* = 3 per group; the data are presented as the mean ± SD; ns not significant; *P < 0.05; and **P < 0.01.

CLIP1 can regulate the function of ECs directly related to the development of coronary artery disease^36^. Additionally, CLIP1 acts as a negative regulator of the TLR4/MyD88 pathway by inducing TIRAP ubiquitination, including downregulating the activity of NF-κB and MAPK^20,37^. LPS can bind to TLR4 as a DAMP and activate the TLR4/MyD88/NF-κB inflammatory pathway^38,39^. Therefore, we used LPS to induce inflammation in HUVECs to investigate the role of CLIP1. In the cell model, TFPI2 overexpression led to the upregulation of inflammatory response-related proteins (IL-1β, TNF-α, HMGB1, p-P65, and p-IkB), and CLIP1 overexpression partially reversed the upregulation of these proteins mediated by TFPI2 (Fig. 5a–d). The detection of inflammatory factors in the cell culture supernatant further confirmed that CLIP1 could rescue the inflammation mediated by TFPI2 (Fig. 5e–g). These data suggest that TFPI2/CLIP1 may regulate the NF-κB inflammatory pathway; therefore, we further investigated the activity of the NF-κB gene using a dual-luciferase reporter gene system. TFPI2 expression increased the activity of the NF-κB reporter gene, whereas CLIP1 reduced the activation activity of TFPI2 on NF-κB (Fig. 5h). CLIP1 overexpression reduced the level of apoptosis caused by TFPI2 (Fig. 5i, j). These data indicate that CLIP1 can rescue the inflammation and apoptosis caused by TFPI2, which mainly involves the NF-κB inflammatory signaling pathway.

### TFPI2 inhibits TIRAP ubiquitination by regulating CLIP1

**Fig. 6:**
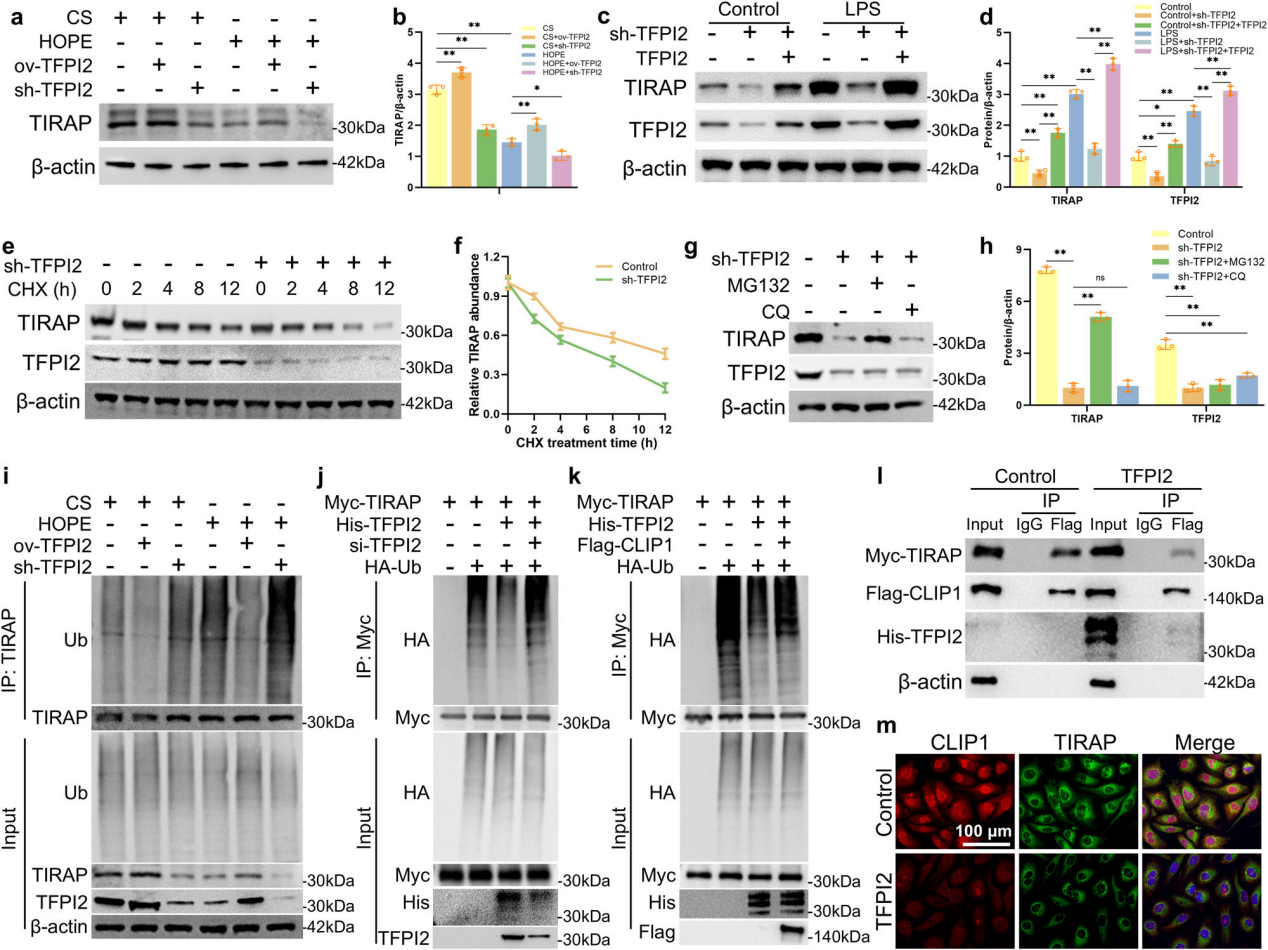
TFPI2 inhibits the ubiquitination of TIRAP by regulating CLIP1. **a**, **b** Western blotting and statistical analyses of the TIRAP protein in the liver tissue of rats infected with ov-TFPI2, sh-TFPI2, or the empty vector AAV8. **c**, **d** Western blotting and statistical analyses of the TIRAP and TFPI2 proteins in HUVECs treated with LPS and transfected with lentivirus of sh-TFPI2, plasmid of TFPI2, or empty vector. **e**, **f** Western blotting of the TIRAP and TFPI2 proteins in HUVECs transfected with sh-TFPI2 or empty vector and treated with CHX for different times and the degradation curves of the TIRAP protein. **g, h** Western blotting and statistical analyses of the TIRAP and TFPI2 proteins in HUVECs transfected with sh-TFPI2 or empty vector and treated with MG132 or chloroquine. **i** Analysis of TFPI2 regulating endogenous TIRAP ubiquitination. Rats were infected with ov-TFPI2, sh-TFPI2, or the empty vector AAV8. Liver tissue protein extracts were tested by CoIP with an anti-TIRAP antibody. **j**, **k** Analysis of TFPI2 and CLIP1 regulating exogenous TIRAP ubiquitination. HEK293T cells were transfected with Myc-tagged TIRAP, Flag-tagged CLIP1, HA-tagged Ub, or si-TFPI2. Cell protein extracts were subjected to CoIP with an anti-Myc antibody. **l** CoIP assays of TIRAP and CLIP1 expression in HEK293T cells with or without TFPI2 overexpression. **m** IF analysis of CLIP1 and TIRAP in HUVECs with or without TFPI2 overexpression. *n* = 3 per group; the data are presented as the mean ± SD; ns not significant; *P < 0.05; and **P < 0.01.

TIRAP acts as an adapter protein, coupling TLR4 and MyD88 in the TLR4/MyD88/NF-κB inflammatory pathway^20,37^. CLIP1 can induce the ubiquitination and degradation of TIRAP, thus negatively regulating the TLR4 inflammatory response^20,37^. We also demonstrated that CLIP1 promoted TIRAP ubiquitination in vitro (Supplementary Fig. 7). We studied the relationship between TFPI2 and TIRAP to explore the effect of TFPI2 on this process. The protein expression level of TIRAP in liver tissue was increased in the CS group and decreased in the HOPE group in a rat model of fatty liver IRI. TFPI2 overexpression increased TIRAP levels, whereas TFPI2 silencing decreased TIRAP levels (Fig. 6a, b). The protein expression of TIRAP and TFPI2 was increased in an LPS-induced cell inflammation model. Furthermore, silencing TFPI2 decreased the level of TIRAP, and after restoring TFPI2 expression, the level of TIRAP significantly increased (Fig. 6c, d). These data suggested that HOPE regulated the expression of TIRAP via TFPI2.

Next, we investigated how TFPI2 regulates TIRAP. We treated HUVECs with the protein synthesis inhibitors cycloheximide (CHX) and sh-TFPI2 and found that silencing TFPI2 shortened the half-life of the endogenous TIRAP protein (Fig. 6e, f). These findings indicated that TFPI2 regulated TIRAP stability. Only the proteasomal inhibitor MG132, not chloroquine (CQ), a lysosomal protein inhibitor, rescued protein degradation (Fig. 6g, h), suggesting that TFPI2 inhibits the degradation of TIRAP in a proteasome-dependent manner. The level of TIRAP ubiquitination was significantly reduced in the CS group and increased in the HOPE group in the animal models. TFPI2 overexpression further reduced the ubiquitination of TIRAP, whereas TFPI2 silencing had the opposite effect (Fig. 6i). These findings indicate that HOPE promotes the ubiquitination of TIRAP by inhibiting TFPI2 expression. In vitro studies also revealed that TFPI2 inhibited the ubiquitination of TIRAP, whereas siTFPI2 promoted its ubiquitination (Fig. 6j). The inhibitory effect of TFPI2 on TIRAP ubiquitination was effectively blocked by CLIP1 (Fig. 6k). Notably, TFPI2 inhibited the interaction between CLIP1 and TIRAP (Fig. 6l). IF analysis further revealed that TFPI2 weakened the colocalization of CLIP1 and TIRAP in HUVECs (Fig. 6m). Therefore, TFPI2 inhibited the ubiquitination of TIRAP by mediating the interaction between TIRAP and CLIP1, whereas HOPE inhibited this process.

### Expression of TFPI2 and CLIP1 in human fatty liver after CS

**Fig. 7:**
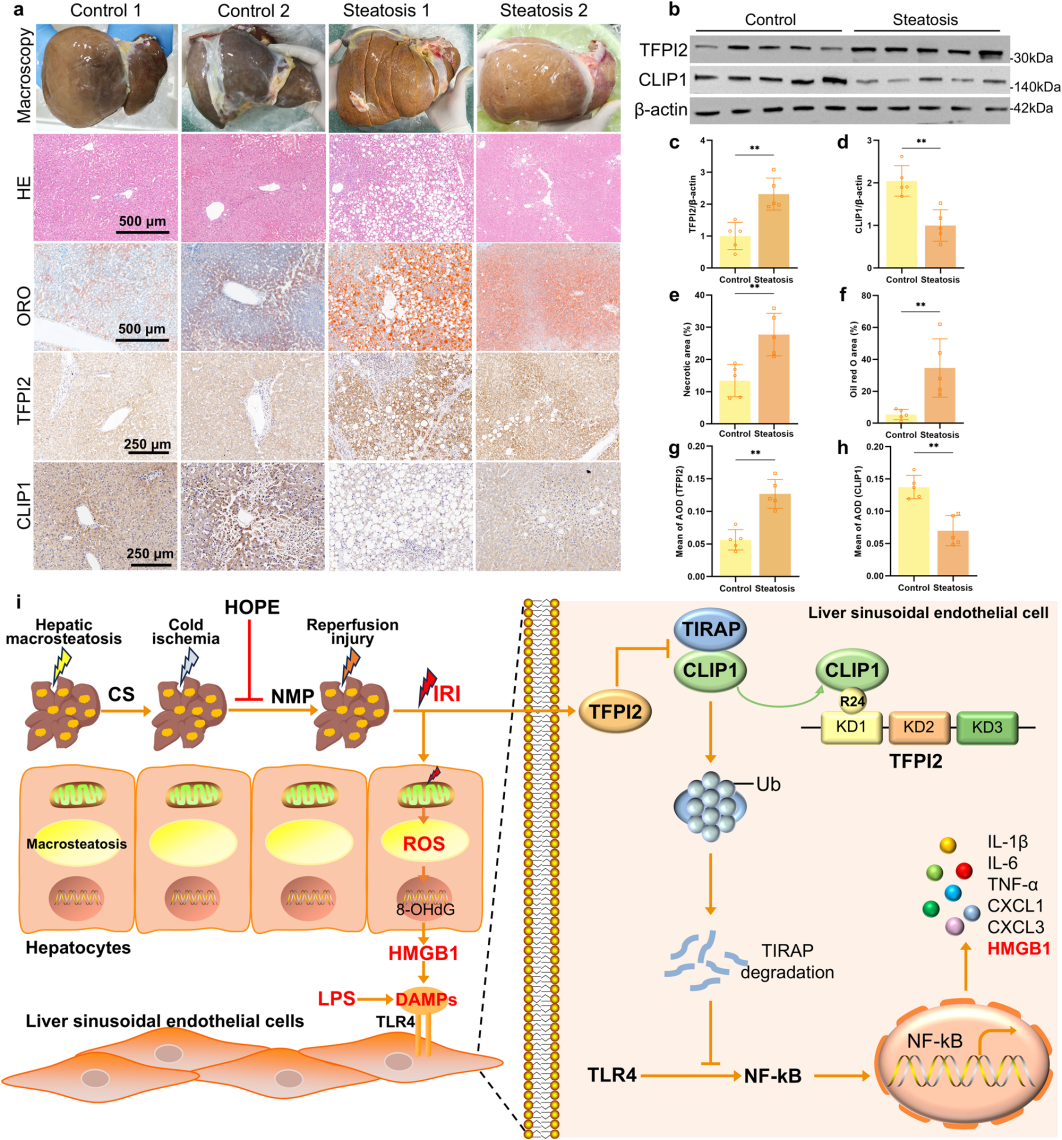
Expression of TFPI2 and CLIP1 in human fatty liver after CS. **a** Macroscopic view of the human liver; HE staining, ORO staining, and IHC staining of TFPI2 and CLIP1. **b–d** Western blotting and statistical analyses of the TFPI2 and CLIP1 proteins in human liver tissue. **e** Analysis of the necrotic area via human liver histology. **f** Analysis of the ORO-positive area in human liver tissue. **g**, **h** Quantitative analysis of IHC staining of TFPI2 and CLIP1 in human liver tissue. *n* = 5 per group; the data are the mean ± SD; ns, not significant; *P < 0.05; and **P < 0.01. **i** Mechanistic scheme. In a rat model of fatty liver, severe steatosis, cold ischemia, and subsequent reperfusion led to the accumulation of ROS in hepatocytes, with the released HMGB1 activating the TLR4/NF-κB inflammatory pathway in ECs. Additionally, R24 of the KD1 domain of TFPI2 directly binds to CLIP1 in ECs, inhibiting the ubiquitination and degradation of TIRAP by CLIP1, thereby activating the TLR4/NF-κB-mediated inflammatory response, resulting in severe IRI. HOPE effectively ameliorates the entire pathological process.

We obtained samples from five cases of nonfatty liver and five cases of fatty liver from clinically discarded livers, all of which underwent 8–24 h of CS before collection to confirm the changes in TFPI2 and CLIP1 molecules in human fatty liver (Fig. 7a and Supplementary Table 3). HE staining revealed that pathological damage in fatty livers worsened to varying degrees after cold preservation compared with that in the control group (Fig. 7a, e). ORO staining showed fatty liver with a significant increase in lipid droplets (Fig. 7a, f). These findings confirmed that fatty livers are more sensitive to cold ischemic injury. Compared with that in the control group, TFPI2 expression was upregulated, and CLIP1 expression was downregulated in fatty livers after CS, as determined via immunohistochemistry (IHC) and Western blotting (Fig. 7a–d, g, h). These findings suggested that cold ischemic injury promoted TFPI2 expression and inhibited CLIP1 expression in fatty livers.

## Discussion

Steatosis can be divided into macrosteatosis and microsteatosis, and these two fatty liver types have different degrees of tolerance to IRI^11,40^. Severe macrosteatosis significantly affects the functional recovery of the transplanted liver and the occurrence of PNF^11^. We used MCD to induce a rat fatty liver model with >60% macrosteatosis to simulate severe macrosteatosis in the liver. First, we found that fatty liver after ischemia experiences a corresponding reperfusion injury, and the degree of injury to the fatty liver after a period of cold ischemia is twice that of normal liver. Second, the levels of cell necrosis, apoptosis, oxidative stress response, and inflammation significantly improved in fatty livers treated with HOPE. Importantly, the enrichment of DEGs in the transcriptome related to inflammatory pathways and inflammatory molecules highlights the significant potential of HOPE to inhibit inflammatory responses in fatty liver IRI. The changes in HMGB1, TLR4, and NF-κB-related molecules suggest that the key downstream pathways regulated by HOPE include the activation of Toll-like receptors. In addition to the obvious changes in inflammation-related genes and pathways, we also observed significant alterations in genes associated with fluid shear stress, lipid metabolism, and immune responses via the GO and KEGG enrichment analyses (Fig. 2a–d, Supplementary Fig. 4). Specifically, mechanical perfusion-induced shear stress on ECs provides a novel perspective for studying IRI^41,42^. Furthermore, lipid metabolism and immune responses are also involved in the regulation of IRI^43,44^. These unexpected findings may offer new insights for enhancing the quality of fatty liver grafts.

The CS time was extended to 24 h to simulate clinical scenarios. Prolonged cold ischemia primarily targets LSECs (manifested as cell body retraction and detachment), with subsequent warm reperfusion leading to almost complete denudation of the LSEC lining^45,46^. Additionally, the excessive accumulation of large lipid droplets within hepatocytes exacerbates ROS damage and results in the release of more DAMPs (such as HMGB1). DAMPs can recognize TLR4 or TLR9 on the EC membrane, activating inflammatory pathways^6,7,47^. This dual insult further aggravates EC injury. Surprisingly, we found that HOPE treatment alleviated the inflammatory response and oxidative stress in fatty liver and significantly mitigated EC damage.

TFPI2 was differentially expressed according to transcriptomic data analysis. The inhibitory activity of the KD1 domain of TFPI2 was greater than that of the other domains, likely owing to the inclusion of all of the structural elements necessary for serine protease inhibition^30,48^. The inhibitory activity of KD1, mediated by R24, decreases the inhibitory activity of TFPI2 by approximately 90% following mutations in R24Q or R24K^48,49^. TFPI2 is synthesized primarily by ECs from different vessels, is secreted into the extracellular matrix, and regulates the activation of matrix metalloproteinases (MMPs) mediated by plasmin and pancreatic enzymes, playing a crucial role in extracellular matrix remodeling^50^. TFPI2 also acts as a tumor suppressor gene to induce tumor cell apoptosis^49,51^. It can regulate kidney function by inducing mesangial cell apoptosis^52,53^. TFPI2 overexpression in ECs can inhibit cell migration and angiogenesis and increase apoptosis^31^. A study on the regulation of liver inflammation revealed that TFPI2 expression in the human liver was mainly observed in LSECs, with a significant increase in TFPI2 expression observed in an LPS-induced mouse model^27^. Our experimental results indicated that TFPI2 expression significantly increased in fatty livers after CS, showing a similar trend to that of inflammatory levels, which could be reversed by HOPE treatment. Furthermore, TFPI2 overexpression significantly promoted the inflammatory response in fatty liver, including activation of the TLR4/NF-κB signaling pathway, whereas inhibiting TFPI2 had the opposite effect. In an LPS-induced HUVEC model of inflammation, TFPI2 plays a significant role in promoting inflammation and apoptosis.

Additionally, CoIP combined with LC‒MS revealed that CLIP1 interacted with TFPI2, with the binding site at R24 of the KD1 domain of TFPI2. Furthermore, CLIP1 exerts a negative regulatory effect on TLR4 by inducing TIRAP ubiquitination (both monoubiquitination and polyubiquitination) and enhancing its proteasomal degradation, consequently diminishing the functionality of the downstream NF-kB and MAPK pathways^20,37^. In this study, CLIP1 mitigated the inflammatory response and cell apoptosis induced by TFPI2. Notably, CLIP1 reduced NF-κB activation and the release of inflammatory factors, including DAMPs (HMGB1), caused by TFPI2. These findings suggest that CLIP1 counteracts the detrimental effects of TFPI2, particularly inflammation.

During TLR4 signal transduction, LPS or DAMPs recognize and bind to TLR4, initiating signaling via TIRAP^38^. TIRAP acts as an adapter, recruiting MyD88 to TLR4 and mediating protein interactions and posttranslational modifications to activate downstream transcription factors^38^. CLIP1 promotes the ubiquitination and degradation of TIRAP, primarily through the MyD88-dependent TLR4 signaling pathway^20^. We found that TFPI2 inhibited the ubiquitination and degradation of TIRAP, whereas CLIP1 enhanced TIRAP ubiquitination. Although CLIP1 lacks traditional ubiquitin ligase domains, such as HECT, RING, and U-box domains, CLIP1 exhibits ubiquitin ligase-like characteristics^20^. Consequently, CLIP1 functions as a scaffold to promote the ubiquitination of the target protein TIRAP. TFPI2 can suppress the ubiquitination and degradation of TIRAP by inhibiting CLIP1, thereby initiating a cascade reaction mediated by TLR4, leading to the activation of downstream NF-κB signaling and the leakage of inflammatory factors. Released HMGB1 in the extracellular space can act as a DAMP to activate the TLR4 signaling pathway, forming a vicious cycle and triggering a cascade of inflammatory responses. Finally, we confirmed increased TFPI2 expression and decreased CLIP1 expression in human liver samples from fatty liver donors after CS, which further supported our findings.

Our study had several limitations. Liver reperfusion after transplantation simulated in vitro with a KHB solution at 37 °C may not fully replicate the in vivo transplantation scenario. Despite maintaining oxygen levels above 500 mmHg in the KHB solution during NMP, the oxygen dissolved in the solution without an oxygen carrier may be insufficient to adequately support metabolism^23,54^. Additionally, prolonged NMP with KHB alone (>2 h) can lead to significant liver damage^55,56^. Therefore, we limited the NMP time to less than 2 h to minimize the damage caused by perfusion while ensuring sufficient time for reperfusion. Because of some constraints, we did not apply HOPE therapy to human liver samples in our clinical experiments.

Overall, we found that HOPE plays an important role in alleviating fatty liver IRI by regulating the TFPI2/CLIP1/TIRAP pathway to suppress the inflammatory response in a rat model of severe fatty liver. During fatty liver IRI, a large amount of ROS is generated in hepatocytes, leading to the leakage of HMGB1 into the extracellular space and the activation of the TLR4/NF-κB inflammatory pathway in ECs. Furthermore, R24 of the KD1 domain of TFPI2 directly binds to CLIP1 within ECs, inhibiting CLIP1-mediated ubiquitination-dependent degradation of TIRAP, thereby activating the TLR4/NF-κB inflammatory response and resulting in severe IRI. Nevertheless, the use of HOPE greatly ameliorated the pathological process (Fig. 7i). Therefore, our findings suggest that targeting the TFPI2/CLIP1/TIRAP signaling pathway with HOPE could be a potential therapeutic strategy for alleviated IRI in fatty liver transplantation.

## Supplementary information

Supplementary Information

Original western blots

## Data Availability

The data of this study are available from the corresponding authors upon reasonable request.

## References

[1] Sousa, D. S. R., Weber, A., Dutkowski, P. & Clavien, P. A. Machine perfusion in liver transplantation. *Hepatology* **76**, 1531–1549 (2022). 35488496 10.1002/hep.32546

[2] Cohen, J. C., Horton, J. D. & Hobbs, H. H. Human fatty liver disease: old questions and new insights. *Science* **332**, 1519–1523 (2011). 21700865 10.1126/science.1204265 PMC3229276

[3] Brenner, C., Galluzzi, L., Kepp, O. & Kroemer, G. Decoding cell death signals in liver inflammation. *J. Hepatol.* **59**, 583–594 (2013). 23567086 10.1016/j.jhep.2013.03.033

[4] Cornide-Petronio, M. E. et al. The effect of high-mobility group box 1 in rat steatotic and nonsteatotic liver transplantation from donors after brain death. *Am. J. Transplant.* **16**, 1148–1159 (2016). 26704922 10.1111/ajt.13560

[5] Jimenez-Castro, M. B. et al. The effect of brain death in rat steatotic and non-steatotic liver transplantation with previous ischemic preconditioning. *J. Hepatol.* **62**, 83–91 (2015). 25111175 10.1016/j.jhep.2014.07.031

[6] Kron, P., Schlegel, A., Mancina, L., Clavien, P. A. & Dutkowski, P. Hypothermic oxygenated perfusion (HOPE) for fatty liver grafts in rats and humans. *J. Hepatol.* **S0168-8278**, 32268–7 (2017). 10.1016/j.jhep.2017.08.028 28870676

[7] Gracia-Sancho, J., Caparros, E., Fernandez-Iglesias, A. & Frances, R. Role of liver sinusoidal endothelial cells in liver diseases. *Nat. Rev. Gastroenterol. Hepatol.* **18**, 411–431 (2021). 33589830 10.1038/s41575-020-00411-3

[8] Jiang, F. et al. Hepatocyte-derived extracellular vesicles promote endothelial inflammation and atherogenesis via microRNA-1. *J. Hepatol.* **72**, 156–166 (2020). 31568800 10.1016/j.jhep.2019.09.014

[9] de Graaf, E. L. et al. Grade of deceased donor liver macrovesicular steatosis impacts graft and recipient outcomes more than the Donor Risk Index. *J. Gastroenterol. Hepatol.* **27**, 540–546 (2012). 21777274 10.1111/j.1440-1746.2011.06844.x

[10] Younossi, Z. M., Marchesini, G., Pinto-Cortez, H. & Petta, S. Epidemiology of nonalcoholic fatty liver disease and nonalcoholic steatohepatitis: implications for liver transplantation. *Transplantation* **103**, 22–27 (2019). 30335697 10.1097/TP.0000000000002484

[11] Liu, Z. et al. Clear mortality gap caused by graft macrosteatosis in Chinese patients after cadaveric liver transplantation. *Hepatobil. Surg. Nutr.* **9**, 739–758 (2020). 10.21037/hbsn.2019.12.02 PMC7720047 33299829

[12] Quesnelle, K. M., Bystrom, P. V. & Toledo-Pereyra, L. H. Molecular responses to ischemia and reperfusion in the liver. *Arch. Toxicol.* **89**, 651–657 (2015). 25566829 10.1007/s00204-014-1437-x

[13] Lu, L. et al. Innate immune regulations and liver ischemia-reperfusion injury. *Transplantation* **100**, 2601–2610 (2016). 27861288 10.1097/TP.0000000000001411 PMC5141614

[14] Zhong, X. et al. TAK242 suppresses the TLR4 signaling pathway and ameliorates DCD liver IRI in rats. *Mol. Med. Rep.* **20**, 2101–2110 (2019). 31257518 10.3892/mmr.2019.10439 PMC6691197

[15] Ma, J. et al. The TNF family member 4-1BBL sustains inflammation by interacting with TLR signaling components during late-phase activation. *Sci. Signal.* **6**, ra87 (2013). 24084649 10.1126/scisignal.2004431 PMC4004444

[16] Rajpoot, S. et al. TIRAP in the mechanism of inflammation. *Front. Immunol.* **12**, 697588 (2021). 34305934 10.3389/fimmu.2021.697588 PMC8297548

[17] Sakaguchi, M. et al. TIRAP, an adaptor protein for TLR2/4, transduces a signal from RAGE phosphorylated upon ligand binding. *PLoS ONE* **6**, e23132 (2011). 21829704 10.1371/journal.pone.0023132 PMC3148248

[18] Baig, M. S. et al. Heterotrimeric complex of p38 MAPK, PKCdelta, and TIRAP is required for AP1 mediated inflammatory response. *Int. Immunopharmacol.* **48**, 211–218 (2017). 28528205 10.1016/j.intimp.2017.04.028

[19] You, X. et al. Macrophage-activating lipopeptide-2 requires Mal and PI3K for efficient induction of heme oxygenase-1. *PLoS ONE* **9**, e103433 (2014). 25077631 10.1371/journal.pone.0103433 PMC4117634

[20] Jakka, P. et al. Cytoplasmic linker protein CLIP170 negatively regulates TLR4 signaling by targeting the TLR adaptor protein TIRAP. *J. Immunol.* **200**, 704–714 (2018). 29222167 10.4049/jimmunol.1601559 PMC5760445

[21] Schlegel, A. et al. Hypothermic oxygenated perfusion protects from mitochondrial injury before liver transplantation. *Ebiomedicine* **60**, 103014 (2020). 32979838 10.1016/j.ebiom.2020.103014 PMC7519249

[22] Zhou, W. et al. Hypothermic oxygenated perfusion inhibits HECTD3-mediated TRAF3 polyubiquitination to alleviate DCD liver ischemia-reperfusion injury. *Cell Death Dis.* **12**, 211 (2021). 33627626 10.1038/s41419-021-03493-2 PMC7904838

[23] He, W. et al. Hypothermic oxygenated perfusion (HOPE) attenuates ischemia/reperfusion injury in the liver through inhibition of the TXNIP/NLRP3 inflammasome pathway in a rat model of donation after cardiac death. *FASEB J*. **32**, 6212–6227 (2018). 10.1096/fj.201800028RR 29870680

[24] Yue, P. et al. Hypothermic oxygenated perfusion attenuates DCD liver ischemia-reperfusion injury by activating the JAK2/STAT3/HAX1 pathway to regulate endoplasmic reticulum stress. *Cell. Mol. Biol. Lett.* **28**, 55 (2023). 37438690 10.1186/s11658-023-00466-5 PMC10337067

[25] Wang, S. et al. Hypothermic oxygenated perfusion ameliorates ischemia-reperfusion injury of fatty liver in mice via Brg1/Nrf2/HO-1 axis. *Artif. Organs* **46**, 229–238 (2022). 34570898 10.1111/aor.14076

[26] Song, D., Li, C., Zhu, M., Chi, S. & Liu, Z. Tracking hepatic ischemia-reperfusion injury in real time with a reversible NIR-IIb fluorescent redox probe. *Angew. Chem. -Int. Ed.* **61**, e202212721 (2022). 10.1002/anie.202212721 36123304

[27] Hisaka, T. et al. Expression of tissue factor pathway inhibitor-2 in murine and human liver regulation during inflammation. *Thromb. Haemost.* **91**, 569–575 (2004). 14983234 10.1160/TH03-06-0358

[28] Zhang, Q. et al. Reduced expression of tissue factor pathway inhibitor-2 contributes to apoptosis and angiogenesis in cervical cancer. *J. Exp. Clin. Cancer Res.* **31**, 1 (2012). 22208663 10.1186/1756-9966-31-1 PMC3314549

[29] George, J., Gondi, C. S., Dinh, D. H., Gujrati, M. & Rao, J. S. Restoration of tissue factor pathway inhibitor-2 in a human glioblastoma cell line triggers caspase-mediated pathway and apoptosis. *Clin. Cancer Res.* **13**, 3507–3517 (2007). 17575213 10.1158/1078-0432.CCR-06-3023 PMC1905856

[30] Chand, H. S., Foster, D. C. & Kisiel, W. Structure, function and biology of tissue factor pathway inhibitor-2. *Thromb. Haemost.* **94**, 1122–1130 (2005). 16411383 10.1160/TH05-07-0509

[31] Ivanciu, L., Gerard, R. D., Tang, H., Lupu, F. & Lupu, C. Adenovirus-mediated expression of tissue factor pathway inhibitor-2 inhibits endothelial cell migration and angiogenesis. *Arterioscler. Thromb. Vasc. Biol.* **27**, 310–316 (2007). 17138934 10.1161/01.ATV.0000254147.89321.cf

[32] Davis, R. J. Signal transduction by the JNK group of MAP kinases. *Cell* **103**, 239–252 (2000). 11057897 10.1016/s0092-8674(00)00116-1

[33] Salomon, A. K. et al. Desmin intermediate filaments and tubulin detyrosination stabilize growing microtubules in the cardiomyocyte. *Basic Res. Cardiol.* **117**, 53 (2022). 36326891 10.1007/s00395-022-00962-3 PMC9633452

[34] Izumi, H. et al. The CLIP1-LTK fusion is an oncogenic driver in non-small-cell lung cancer. *Nature* **600**, 319–323 (2021). 34819663 10.1038/s41586-021-04135-5 PMC8687755

[35] Hu, Y. et al. Tension of plus-end tracking protein Clip170 confers directionality and aggressiveness during breast cancer migration. *Cell Death Dis.* **13**, 856 (2022). 36209218 10.1038/s41419-022-05306-6 PMC9547975

[36] Cho, H. et al. Long noncoding RNA ANRIL regulates endothelial cell activities associated with coronary artery disease by up-regulating CLIP1, EZR, and LYVE1 genes. *J. Biol. Chem.* **294**, 3881–3898 (2019). 30655286 10.1074/jbc.RA118.005050 PMC6422082

[37] Murugan, S., Jakka, P., Namani, S., Mujumdar, V. & Radhakrishnan, G. The neurosteroid pregnenolone promotes degradation of key proteins in the innate immune signaling to suppress inflammation. *J. Biol. Chem.* **294**, 4596–4607 (2019). 30647133 10.1074/jbc.RA118.005543 PMC6433066

[38] Fu, Y. J. et al. Baicalin prevents LPS-induced activation of TLR4/NF-kappaB p65 pathway and inflammation in mice via inhibiting the expression of CD14. *Acta Pharmacol. Sin.* **42**, 88–96 (2021). 32457419 10.1038/s41401-020-0411-9 PMC7921675

[39] Hu, X. et al. Tacrolimus alleviates LPS-induced AKI by inhibiting TLR4/MyD88/NF-kappaB signalling in mice. *J. Cell. Mol. Med.* **26**, 507–514 (2022). 34889045 10.1111/jcmm.17108 PMC8743665

[40] Salvi, M. et al. Fully automated quantitative assessment of hepatic steatosis in liver transplants. *Comput. Biol. Med.* **123**, 103836 (2020). 32658781 10.1016/j.compbiomed.2020.103836

[41] Beijert, I. et al. Endothelial dysfunction in steatotic human donor livers: a pilot study of the underlying mechanism during subnormothermic machine perfusion. *Transplant. Direct* **4**, e345 (2018). 29796416 10.1097/TXD.0000000000000779 PMC5959347

[42] Wang, C., Ren, Y. & Jiang, W. A novel perspective on the mechanisms of ischemia-reperfusion injury: changes in fluid shear stress. *Asian J. Surg.* **47**, 2373–2374 (2024). 38262792 10.1016/j.asjsur.2024.01.065

[43] Li, C. et al. Gp78 deficiency in hepatocytes alleviates hepatic ischemia-reperfusion injury via suppressing ACSL4-mediated ferroptosis. *Cell Death Dis.* **14**, 810 (2023). 38065978 10.1038/s41419-023-06294-x PMC10709349

[44] Tang, Q., Dong, C. & Sun, Q. Immune response associated with ischemia and reperfusion injury during organ transplantation. *Inflamm. Res.* **71**, 1463–1476 (2022). 36282292 10.1007/s00011-022-01651-6 PMC9653341

[45] Guixe-Muntet, S. et al. Cross-talk between autophagy and KLF2 determines endothelial cell phenotype and microvascular function in acute liver injury. *J. Hepatol.* **66**, 86–94 (2017). 27545498 10.1016/j.jhep.2016.07.051

[46] Stolz, D. B. et al. Sinusoidal endothelial cell repopulation following ischemia/reperfusion injury in rat liver transplantation. *Hepatology* **46**, 1464–1475 (2007). 17929236 10.1002/hep.21887 PMC2190086

[47] Tang, S. P. et al. Reactive oxygen species induce fatty liver and ischemia-reperfusion injury by promoting inflammation and cell death. *Front. Immunol.* **13**, 870239 (2022). 35572532 10.3389/fimmu.2022.870239 PMC9098816

[48] Chand, H. S., Schmidt, A. E., Bajaj, S. P. & Kisiel, W. Structure–function analysis of the reactive site in the first Kunitz-type domain of human tissue factor pathway inhibitor-2. *J. Biol. Chem.* **279**, 17500–17507 (2004). 14970225 10.1074/jbc.M400802200

[49] Kempaiah, P. & Kisiel, W. Human tissue factor pathway inhibitor-2 induces caspase-mediated apoptosis in a human fibrosarcoma cell line. *Apoptosis* **13**, 702–715 (2008). 18401718 10.1007/s10495-008-0207-8

[50] Guan, G., Xie, J., Dai, Y. & Han, H. TFPI2 suppresses the interaction of TGF-beta2 pathway regulators to promote endothelial–mesenchymal transition in diabetic nephropathy. *J. Biol. Chem.* **298**, 101725 (2022). 35157852 10.1016/j.jbc.2022.101725 PMC8914548

[51] Sierko, E., Wojtukiewicz, M. Z. & Kisiel, W. The role of tissue factor pathway inhibitor-2 in cancer biology. *Semin. Thromb. Hemost.* **33**, 653–659 (2007). 18000791 10.1055/s-2007-991532

[52] Lin, Y. F. et al. Recombinant tissue factor pathway inhibitor induces apoptosis in cultured rat mesangial cells via its Kunitz-3 domain and C-terminal through inhibiting PI3-kinase/Akt pathway. *Apoptosis* **12**, 2163–2173 (2007). 17885802 10.1007/s10495-007-0136-y

[53] Liang, W. et al. Peptide corresponding to the C terminus of tissue factor pathway inhibitor inhibits mesangial cell proliferation and activation in vivo. *Peptides* **30**, 2330–2336 (2009). 19720104 10.1016/j.peptides.2009.08.016

[54] Zeng, X. et al. Hypothermic oxygenated machine perfusion alleviates donation after circulatory death liver injury through regulating p-selectin-dependent and -independent pathways in mice. *Transplantation* **103**, 918–928 (2019). 31033856 10.1097/TP.0000000000002621

[55] Ferrigno, A., Richelmi, P. & Vairetti, M. Troubleshooting and improving the mouse and rat isolated perfused liver preparation. *J. Pharmacol. Toxicol. Methods* **67**, 107–114 (2013). 23079697 10.1016/j.vascn.2012.10.001

[56] Gores, G. J., Kost, L. J. & Larusso, N. F. The isolated perfused rat liver: conceptual and practical considerations. *Hepatology* **6**, 511–517 (1986). 3519420 10.1002/hep.1840060331

