# Supplementary material for: Hypothermic oxygenated perfusion inhibits CLIP1-mediated TIRAP ubiquitination via TFPI2 to reduce ischemia‒reperfusion injury of the fatty liver
Source: Exp Mol Med. 2024 Dec 2;56(12):2588–601. doi: 10.1038/s12276-024-01350-8 (PMC11671533; doi:10.1038/s12276-024-01350-8)
Supplement: Supplementary file 1 — Supplementary Information [file 12276_2024_1350_MOESM1_ESM.pdf]

Supplementary information

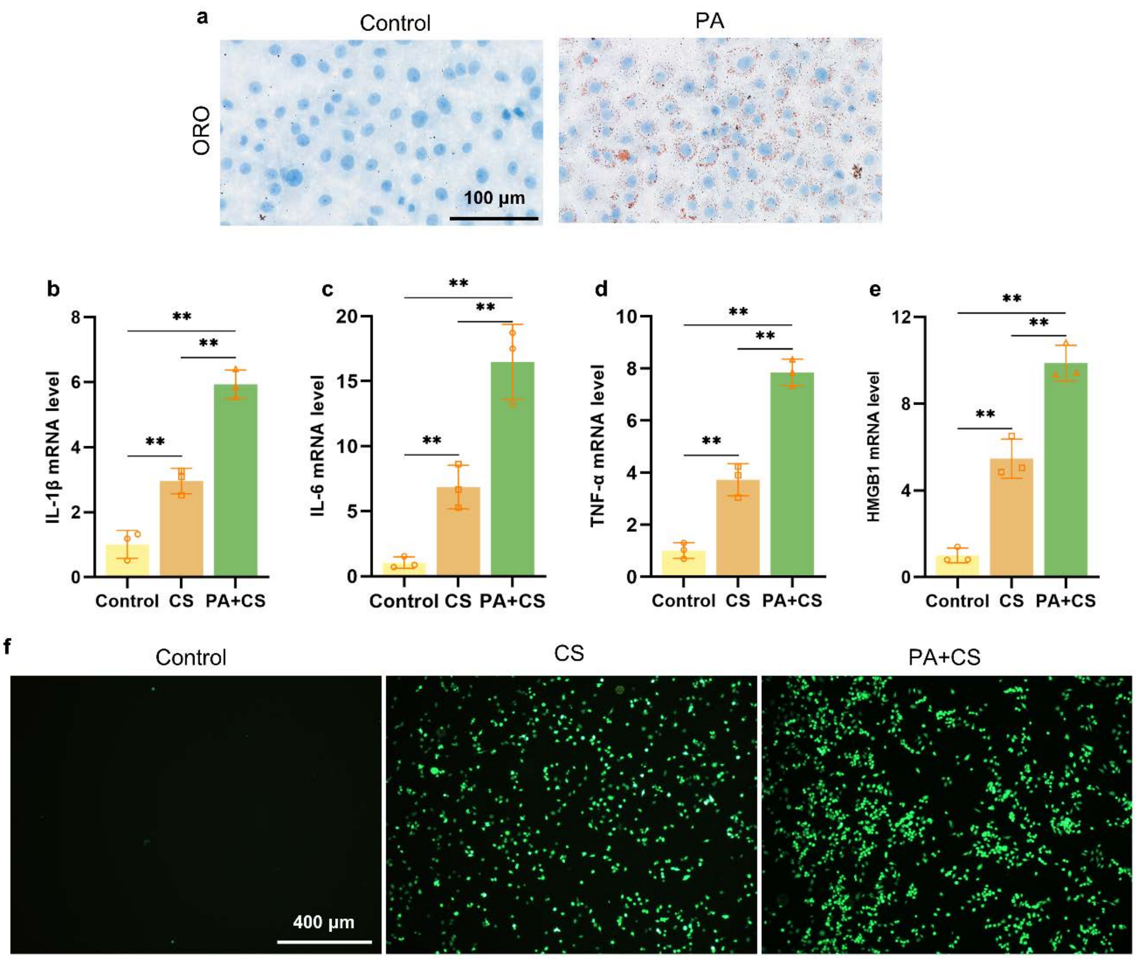

Supplementary Fig. 1 Steatosis BRL-3A cells showed increased sensitivity to IRI. a ORO staining of BRL-3A cells. Cell steatosis was induced by palmitic acid (PA). **b-e** mRNA levels of IL-1β, IL-6, TNF-α, and HMGB1 in BRL-3A cells were detected by PCR. Cells in CS group were stored at 4 ℃ with HTK solution for 12 h, then cultured at 37 ℃ with normal medium for 2 h. The cells in PA+CS group were induced steatosis by PA, and the other operations were the same as those in CS group. **f** ROS staining of BRL-3A cells. n = 3 per group. Data are mean ± SD, ns, no significance, $^{*}P < 0.05$ and $^{**}P < 0.01$.

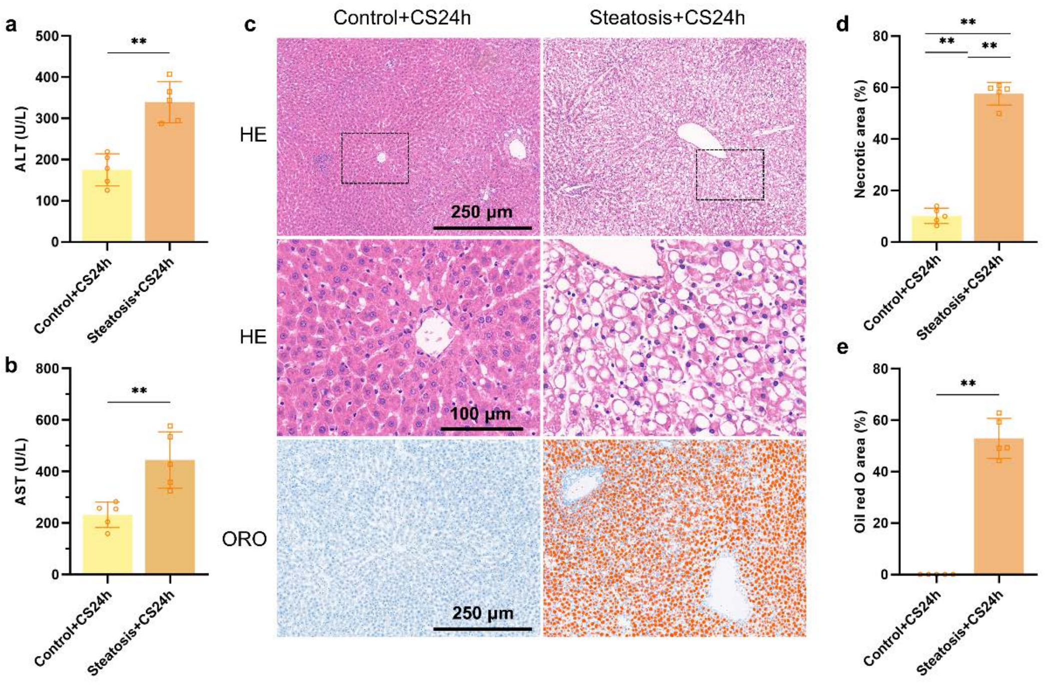

Supplementary Fig. 2 Rat fatty liver showed increased sensitivity to IRI. Control+CS group: the livers of rats with normal diet were stored at 4℃ for 24 h with HTK solution and then treated with KHB solution for 2 h NMP. Steatosis+CS group: the livers of rats with MCD diet were stored at 4 ℃ for 24 h with HTK solution and then treated with KHB solution for 2 h NMP. **a-b** The concentration of ALT and AST in the perfusate. **c** HE staining and ORO staining of liver tissue. **d** Histological analysis of necrotic area of the liver. **e** ORO positive area analysis of liver tissue. n = 5 per group. Data are mean ± SD, ns, no significance, **P < 0.05* and ***P < 0.01*.

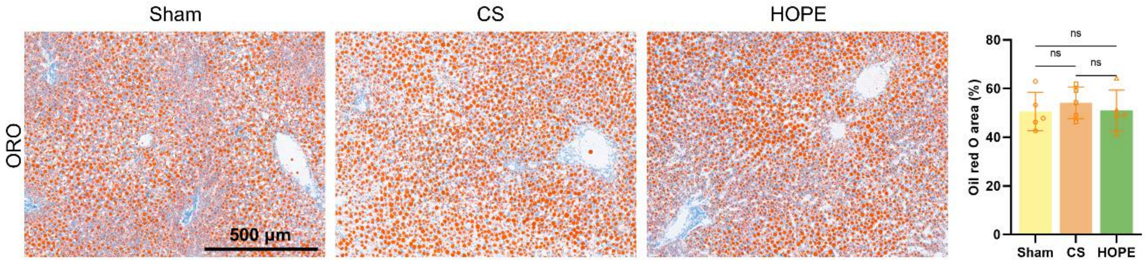

Supplementary Fig. 3 ORO staining and statistical analysis of liver tissue. n = 5 per group. Data are mean ± SD, ns, no significance, *$P < 0.05$* and **$P < 0.01$*.

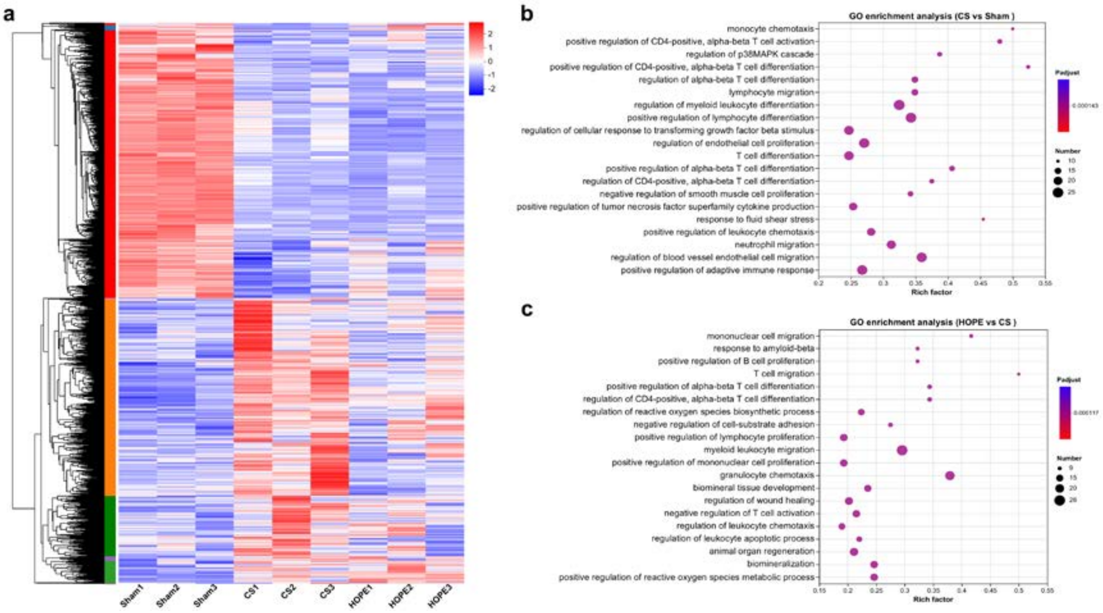

Supplementary Fig. 4 a Heat map of liver DEGs. **b, c** GO enrichment analysis of liver DEGs. n = 3 per group.

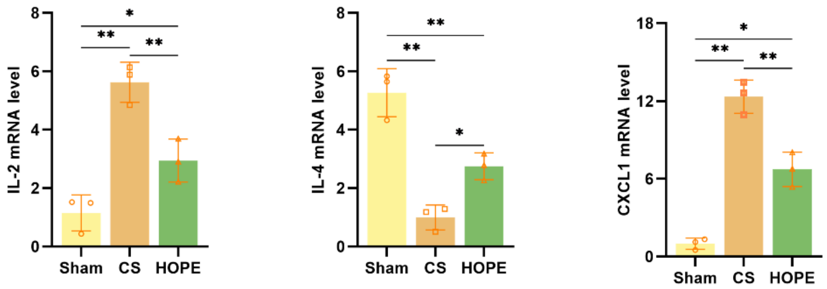

Supplementary Fig. 5 RT-qPCR was used to detect IL-2, IL-4, and CXCL1 mRNA levels in liver tissue. n = 3 per group. Data are mean ± SD, ns, no significance, **P < 0.05* and ***P < 0.01*.

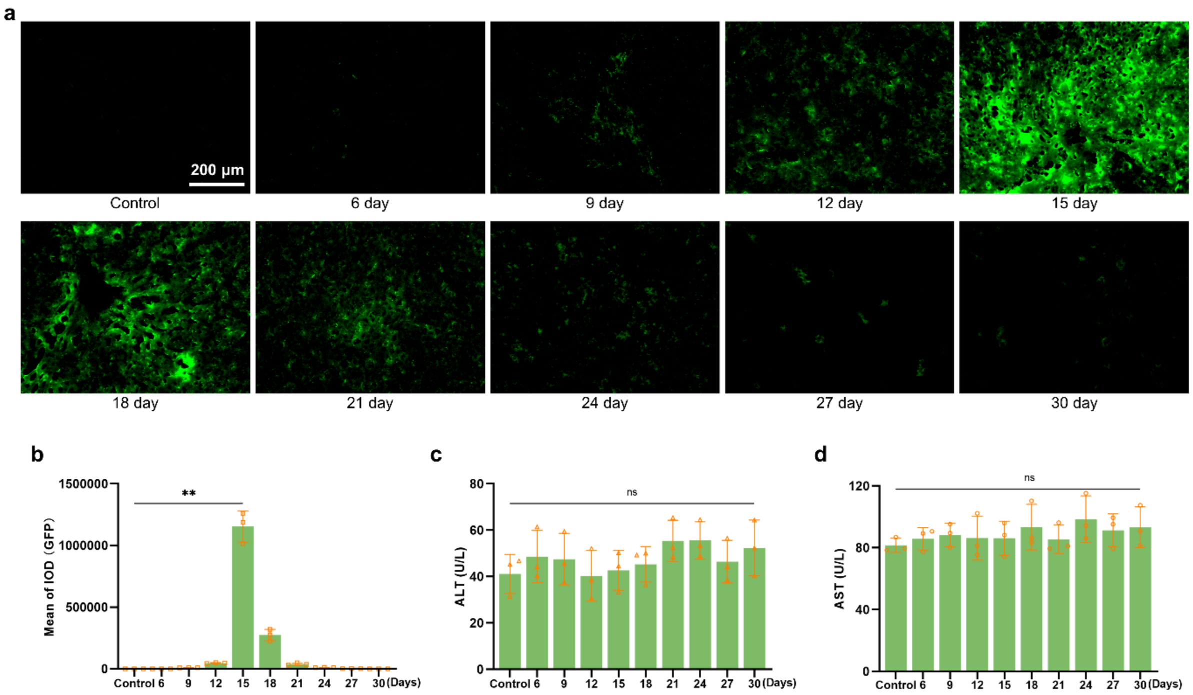

Supplementary Fig. 6 Validation of AAV8 virus transfection efficiency. a Detection of GFP fluorescence. **b** IOD analysis of GFP. **c-d** ALT and AST levels in serum. n = 3 per group. Data are mean ± SD, ns, no significance, **P < 0.05* and ***P < 0.01*.

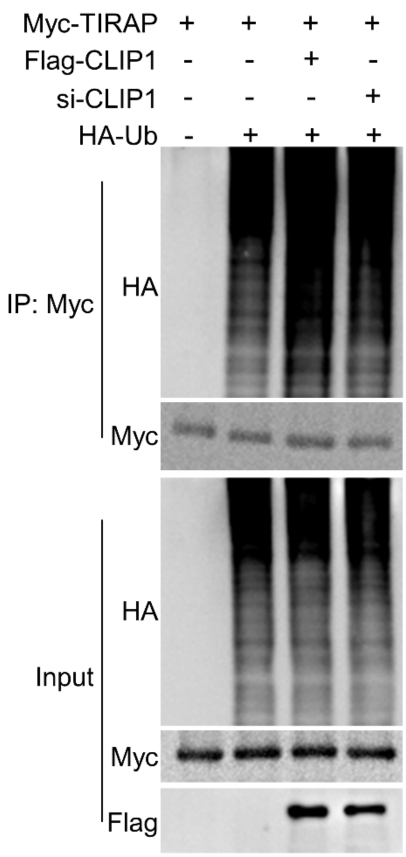

Supplementary Fig. 7 Analysis of CLIP1 regulating exogenous TIRAP ubiquitination. HEK293T cells were transfected with Myc-tagged TIRAP, Flag-tagged CLIP1, HA-tagged Ub, and si-CLIP1. Cell protein extracts were tested by COIP with anti-Myc antibody. n = 3 per group. Data are mean ± SD, ns, no significance, $^{*}P < 0.05$ and $^{**}P < 0.01$.

Supplementary Table 1. Primer and shRNA sequences of the target genes.

Gene name	sequences (5′-3′)
**qRT-PCR primers**	
IL-1β-F	AGCTTCAGGAAGGCAGTGTC
IL-1β-R	TCAGACAGCACGAGGCATTT
IL-2-F	TGTCCTCCTTGTCAACAGCG
IL-2-R	AATTCTGTGGCCTGCTTGGG
IL-4-F	GTACCGGGAACGGTATCCAC
IL-4-R	GTGAGTTCAGACCGCTGACA
IL-6-F	ACAAGTCCGGAGAGGAGACT
IL-6-R	ACAGTGCATCATCGCTGTTC
IL-10-F	TCCCTGGGAGAGAAGCTGAA
IL-10-R	GTAGATGCCGGGTGGTTCAA
TNF-α-F	AAGCTGTCTTCAGGCCAACA
TNF-α-R	CCCGTAGGGCGATTACAGTC
HMGB1-F	AACAACACTTGGTGCTGGGC
HMGB1-R	TCCTCCCAGGGCTTAAGAGAA
CXCL1-F	ACCCAAACCGAAGTCATAGCC
CXCL1-R	GACGCCATCGGTGCAATCTA
CXCL3-F	CCAGACAGAAGTCATAGCCACT
CXCL3-R	GATGGATCGCTGCTCTGCTT

TFPI2-F	CTCGCTTCAGTATCTGCCCA
TFPI2-R	TTCGCAGAGTTTCCGACTGT
β-Actin-F	ACCCGCGAGTACAACCTTCT
β-Actin-R	GCCGTGTTCAATGGGGTACT
**shRNA sequences**	
sh-TFPI2[1]	CCGGATTGAGAACAGGTTT

Supplementary Table 2. The candidate proteins in IP group by MS analysis.

Name	Description	Mw(kDa)	Score
Clip1	CAP-Gly domain-containing linker protein	157.343	323.31
Klhdc4	Kelch domain-containing 4	58.77	41.774
H1-5	Histone H1.5	22.649	41.65
Bltp3b	Similar to CG31653-PA, isoform CRA_a	162.892	34.517
Arl6ip4	ADP-ribosylation factor-like protein 6-interacting protein 4	25.769	34.196

Supplementary Table 3. Basic information of 10 cases of abandoned livers and donors.

Group	Age (y)	gender	steatosis	CS (h)
Control 1	58	Male	none	24
Control 2	80	Male	none	24
Control 3	4	Female	none	8
Control 4	65	Male	none	24
Control 5	63	Male	none	12
Steatosis 1	39	Male	severe	8
Steatosis 2	60	Female	mild	16
Steatosis 3	60	Female	moderate	8
Steatosis 4	66	Female	mild	24
Steatosis 5	52	Male	mild	12

Steatosis is classified as mild (10-30% macrosteatosis), moderate (30-50% macrosteatosis), and severe (> 50% macrosteatosis).

References

1. Yan, W. et al. MBD3 promotes hepatocellular carcinoma progression and metastasis through negative regulation of tumour suppressor TFPI2. *Br. J. Cancer* **127**, 612-623 (2022).
