# Supplementary material for: Hypothermic oxygenated perfusion inhibits CLIP1-mediated TIRAP ubiquitination via TFPI2 to reduce ischemia‒reperfusion injury of the fatty liver
Source: Exp Mol Med. 2024 Dec 2;56(12):2588–601. doi: 10.1038/s12276-024-01350-8 (PMC11671533; doi:10.1038/s12276-024-01350-8)
Supplement: Supplementary file 2 — Original western blots [file 12276_2024_1350_MOESM2_ESM.doc]

**Fig. 3a**

TFPI2 β-actin


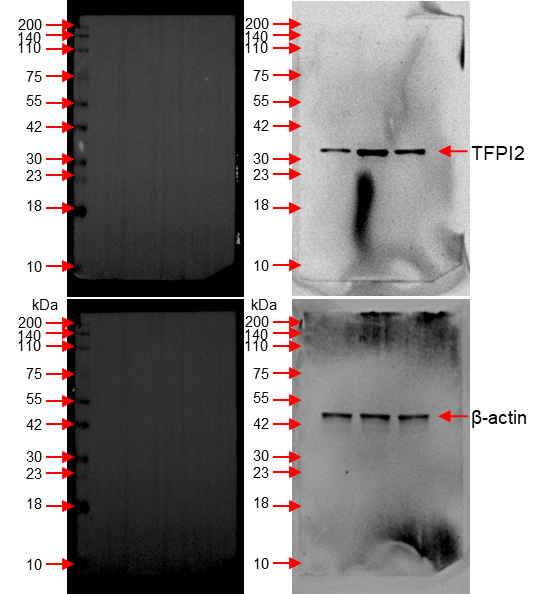


**Fig. 3i**

IL-1β


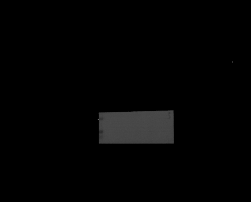

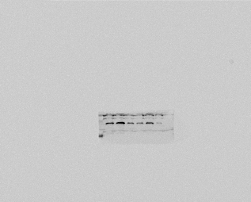


42 kDa

30 kDa

23 kDa

42 kDa

30 kDa

23 kDa

p-P65


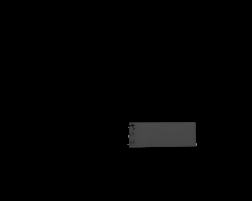

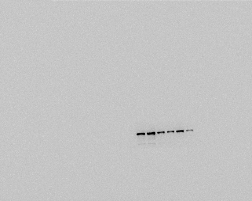


75 kDa

55 kDa

42 kDa

75 kDa

55 kDa

42 kDa

P65


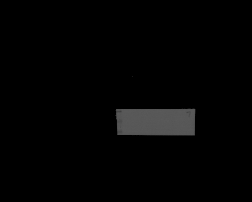

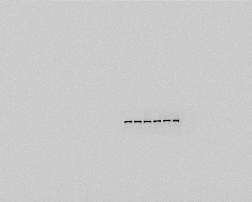


75 kDa

55 kDa

42 kDa

75 kDa

55 kDa

42 kDa

p-ikB


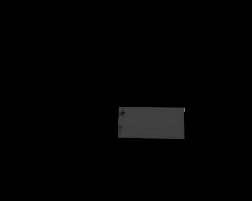

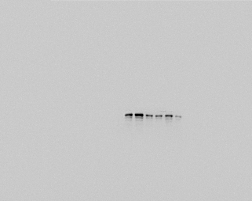


42 kDa

30 kDa

23 kDa

42 kDa

30 kDa

23 kDa

ikB


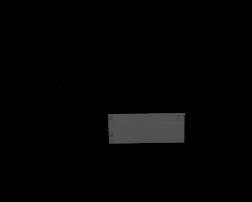

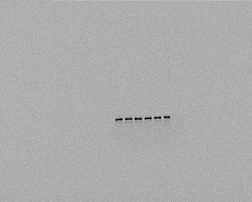


42 kDa

30 kDa

23 kDa

42 kDa

30 kDa

23 kDa

HMGB1


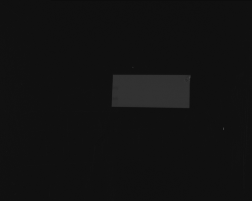

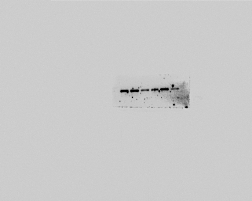


42 kDa

30 kDa

23 kDa

18 kDa

42 kDa

30 kDa

23 kDa

18 kDa

TNF-α


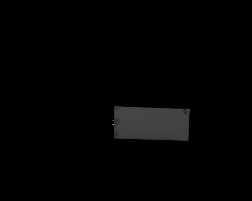

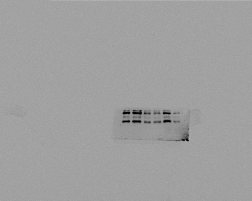


42 kDa

30 kDa

23 kDa

18 kDa

42 kDa

30 kDa

23 kDa

18 kDa

TFPI2


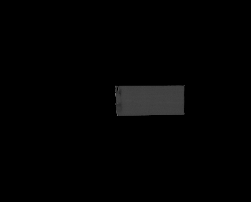

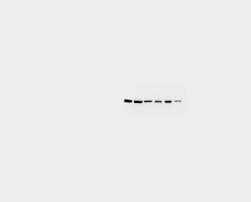


42 kDa

30 kDa

23 kDa

42 kDa

30 kDa

23 kDa

TLR4


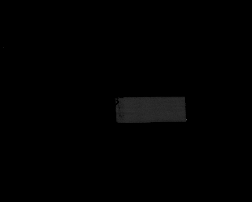

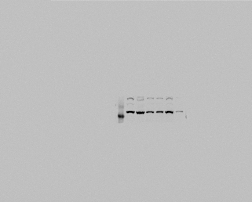


140 kDa

100 kDa

75 kDa

140 kDa

100 kDa

75 kDa

β-actin


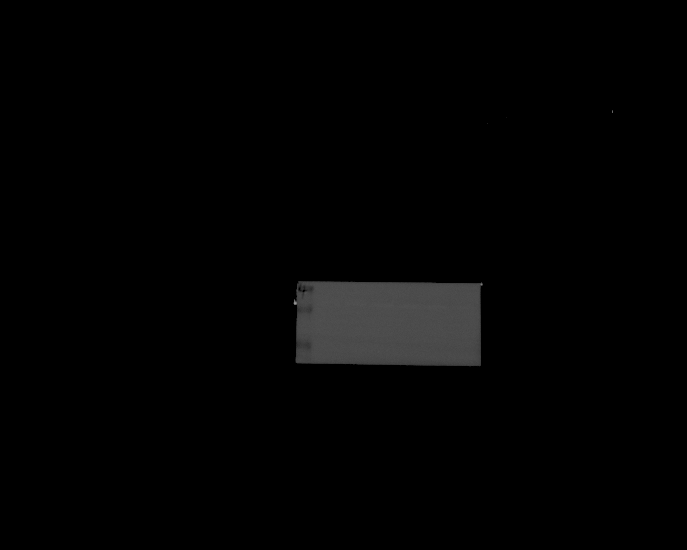

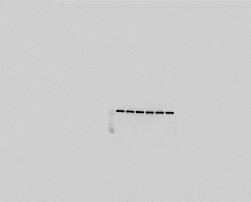


55 kDa

42 kDa

30 kDa

55 kDa

42 kDa

30 kDa

**Fig. 4c**

IP-TFPI2: TFPI2 IP-TFPI2: CLIP1


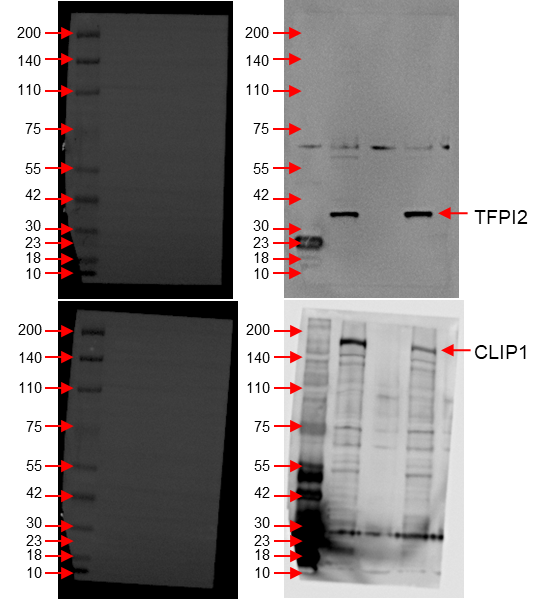


IP-CLIP1: TFPI2 IP-CLIP1: CLIP1


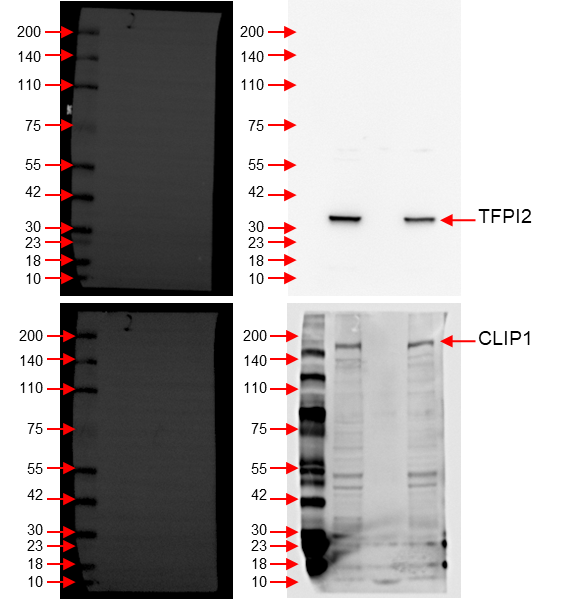


**Fig. 4e**

TFPI2 β-actin


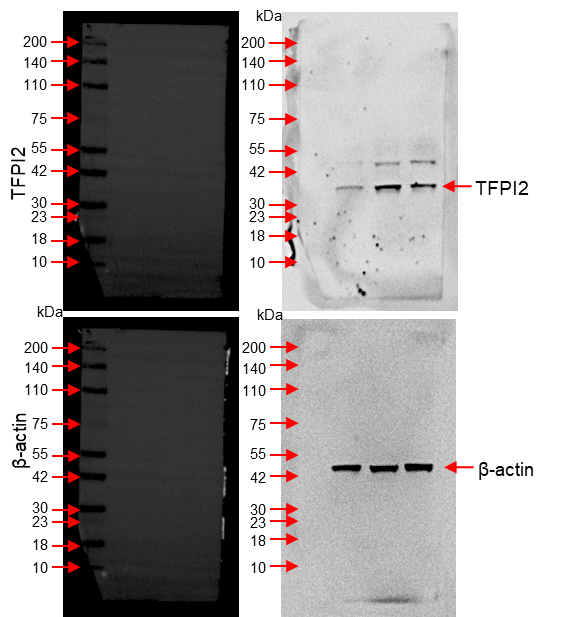


CLIP1 β-actin


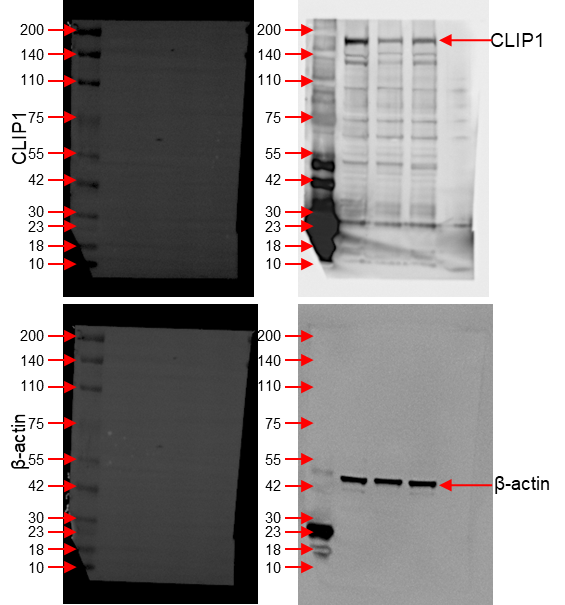


**Fig. 4g**

CLIP1


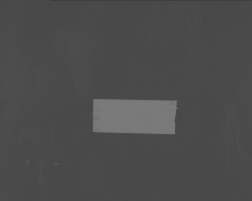

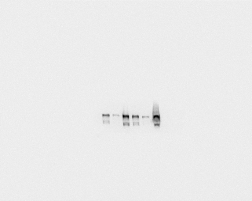


140 kDa

200 kDa

110 kDa

200 kDa

110 kDa

140 kDa

β-actin


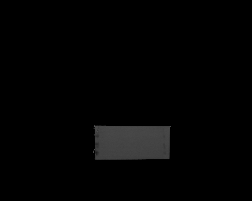

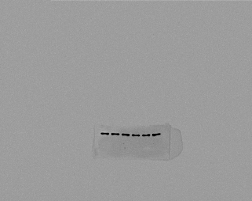


30 kDa

42 kDa

30 kDa

42 kDa

**Fig. 4j**

IP-Flag: Flag


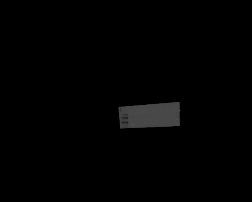

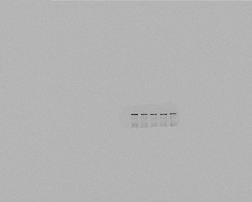


200 kDa

110 kDa

140 kDa

200 kDa

110 kDa

140 kDa

IP-Flag: His


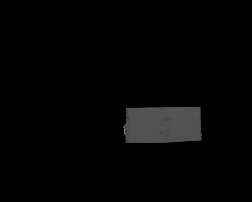

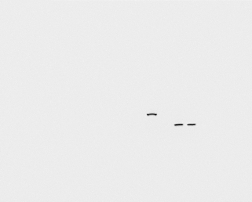

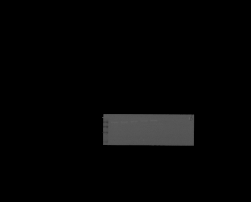

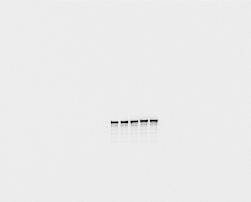


30 kDa

42 kDa

18 kDa

42 kDa

23 kDa

30 kDa

23 kDa

18 kDa

Input: Flag

200 kDa

110 kDa

140 kDa

200 kDa

110 kDa

140 kDa

Input: His


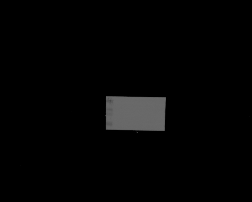

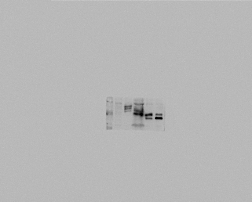


42 kDa

18 kDa

23 kDa

30 kDa

42 kDa

18 kDa

23 kDa

30 kDa

**Fig. 4k**

IP-Flag: Flag IP-Flag: His


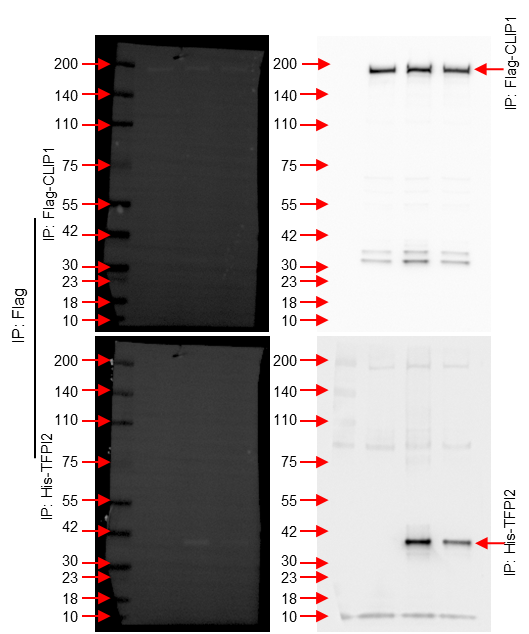


Input: Flag Input: His


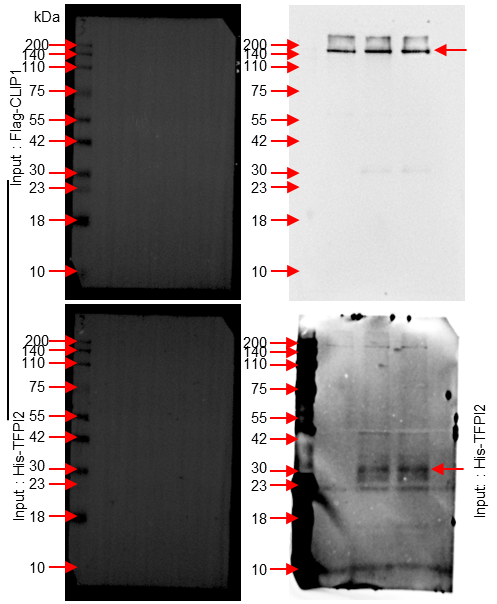


**Fig. 5a**

TFPI2


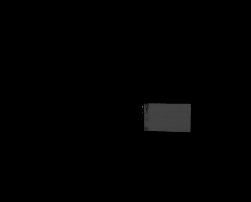

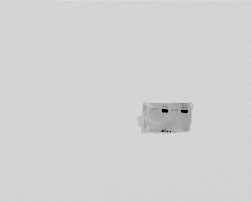

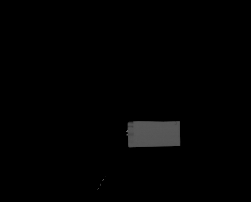

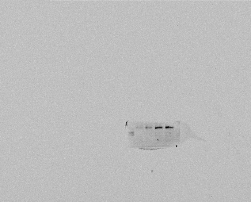


42 kDa

23 kDa

30 kDa

42 kDa

23 kDa

30 kDa

CLIP1

140 kDa

200 kDa

140 kDa

200 kDa

IL-1β


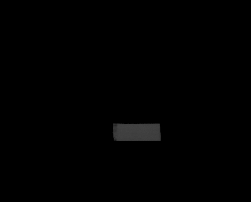

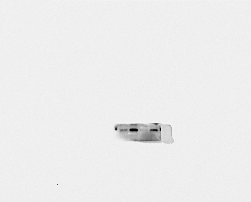

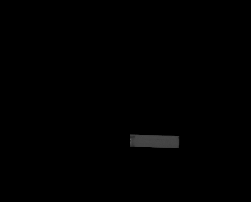

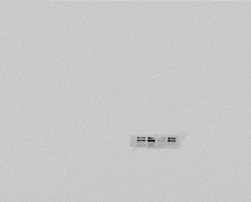

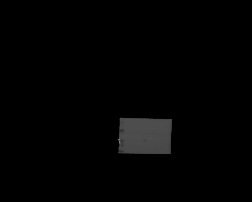

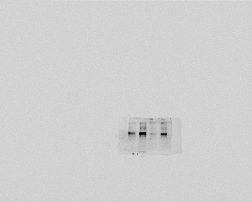

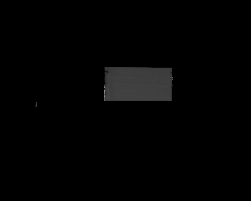

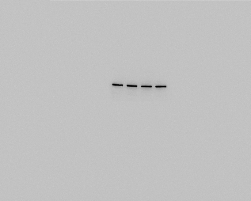


30 kDa

42 kDa

30 kDa

42 kDa

TNF-α

23 kDa

30 kDa

23 kDa

30 kDa

HMGB1

18 kDa

23 kDa

30 kDa

18 kDa

23 kDa

30 kDa

P65

42 kDa

55 kDa

75 kDa

42 kDa

55 kDa

75 kDa

p-p65


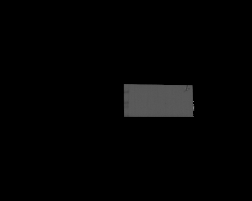

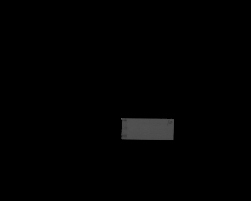

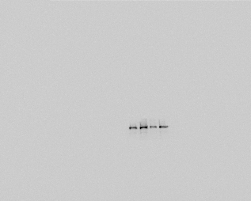

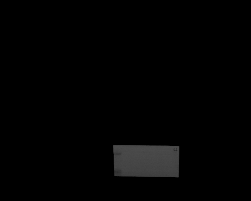

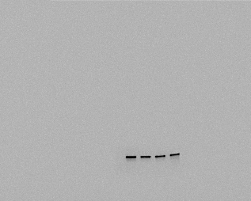

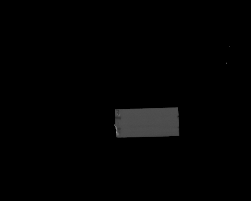

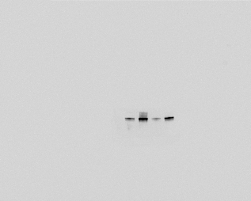

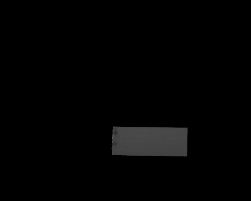

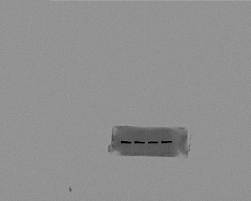

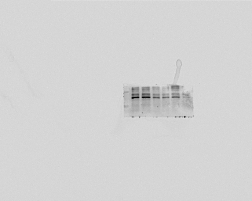

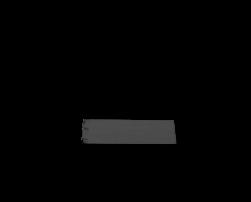

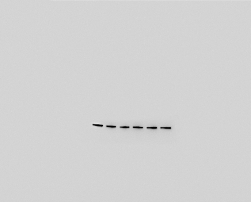


42 kDa

55 kDa

75 kDa

42 kDa

55 kDa

75 kDa

Ikb

30 kDa

42 kDa

30 kDa

42 kDa

p-ikb

30 kDa

42 kDa

30 kDa

42 kDa

β-actin

42 kDa

55 kDa

42 kDa

55 kDa

**Fig. 6a**

TIRAP

30 kDa

42 kDa

23 kDa

42 kDa

23 kDa

30 kDa

β-actin

42 kDa

30 kDa

42 kDa

30 kDa

**Fig. 6c**

TIRAP


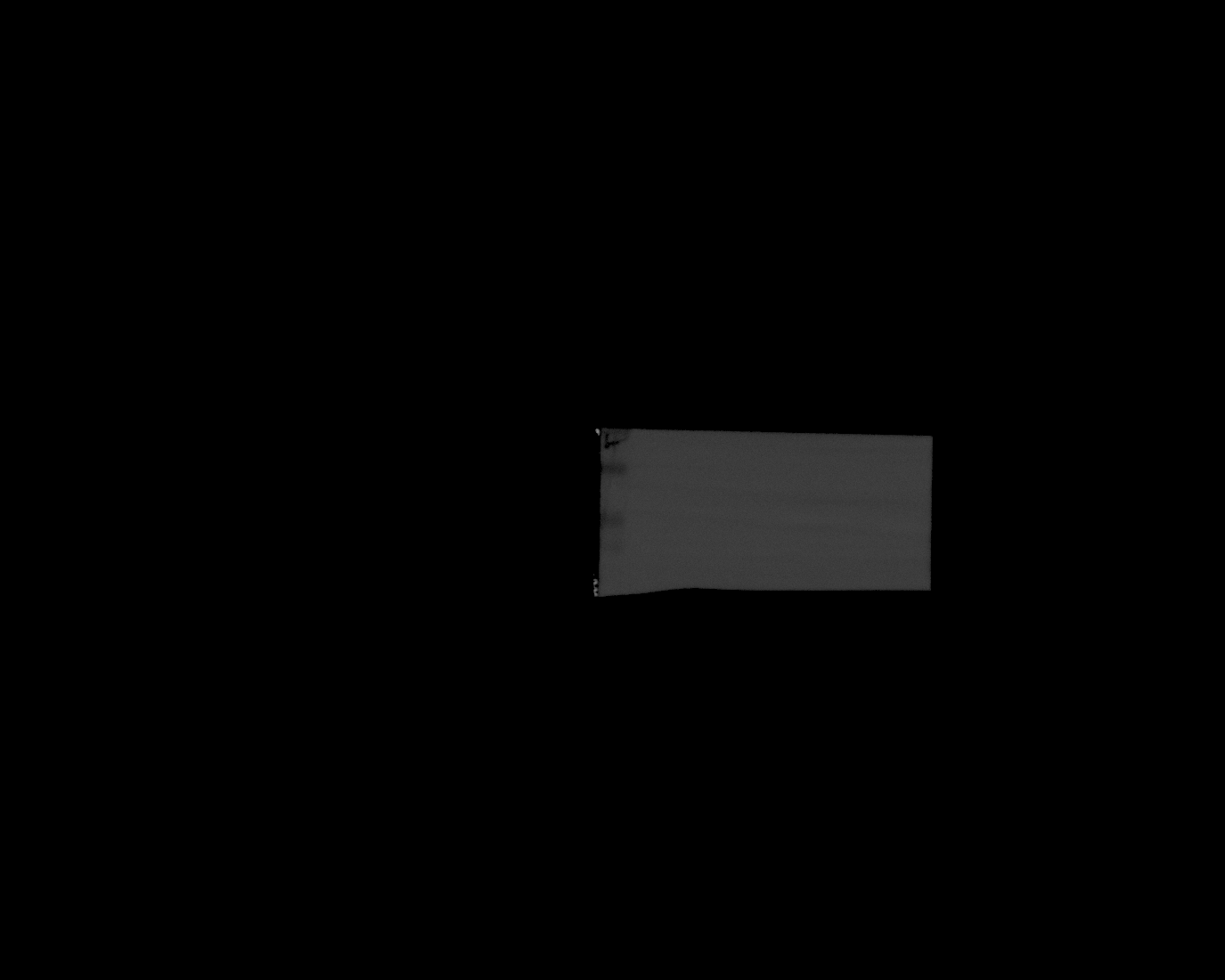

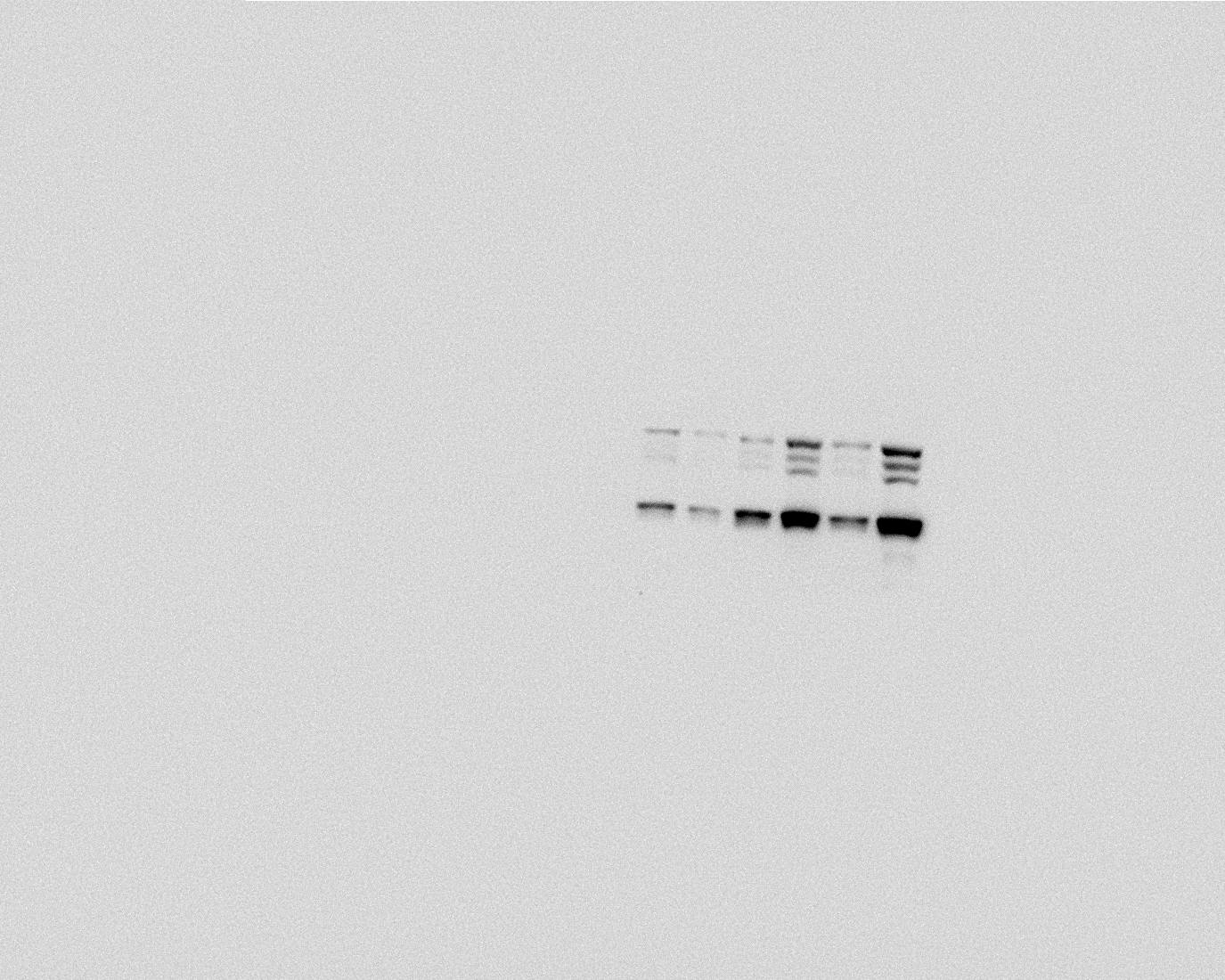

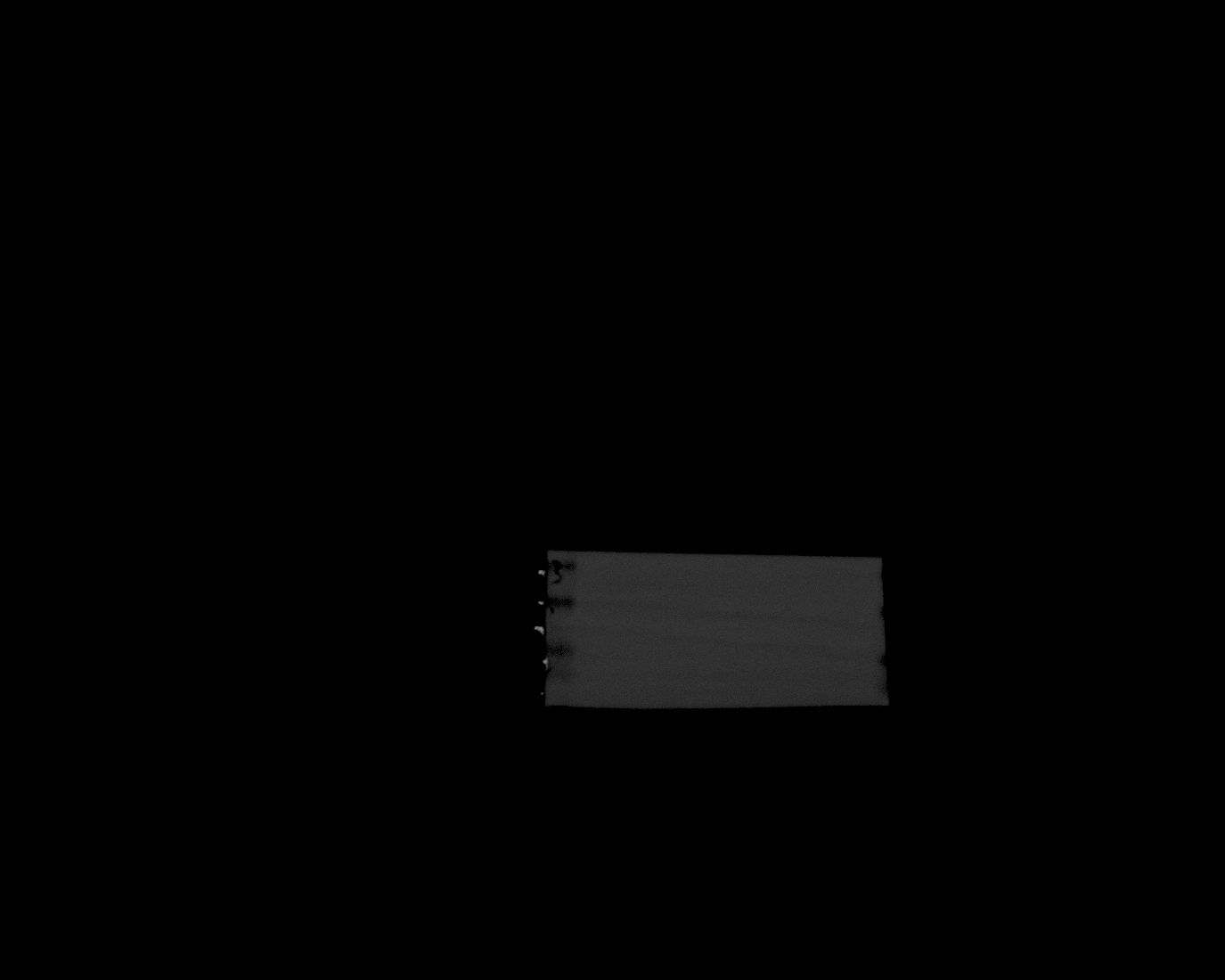

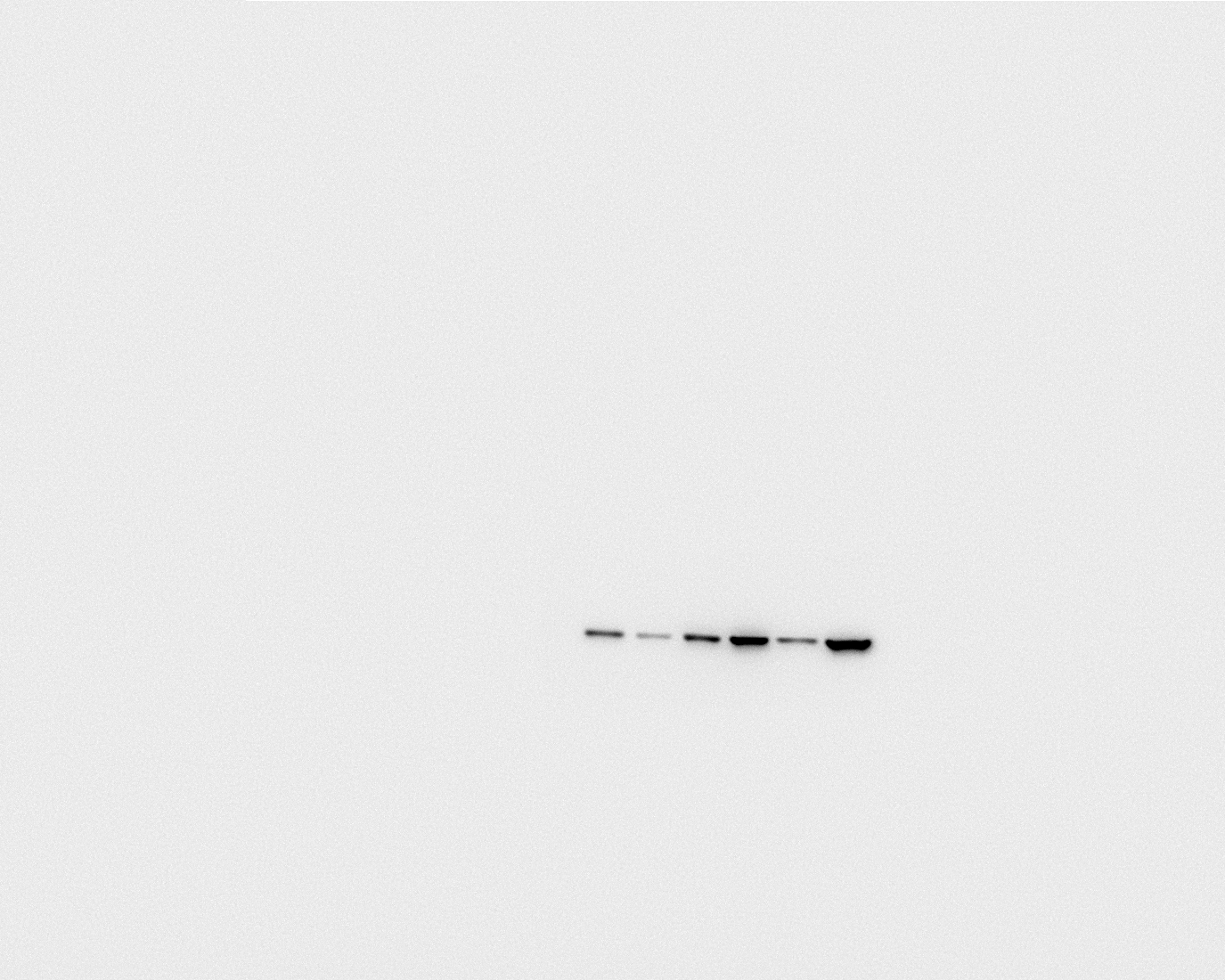

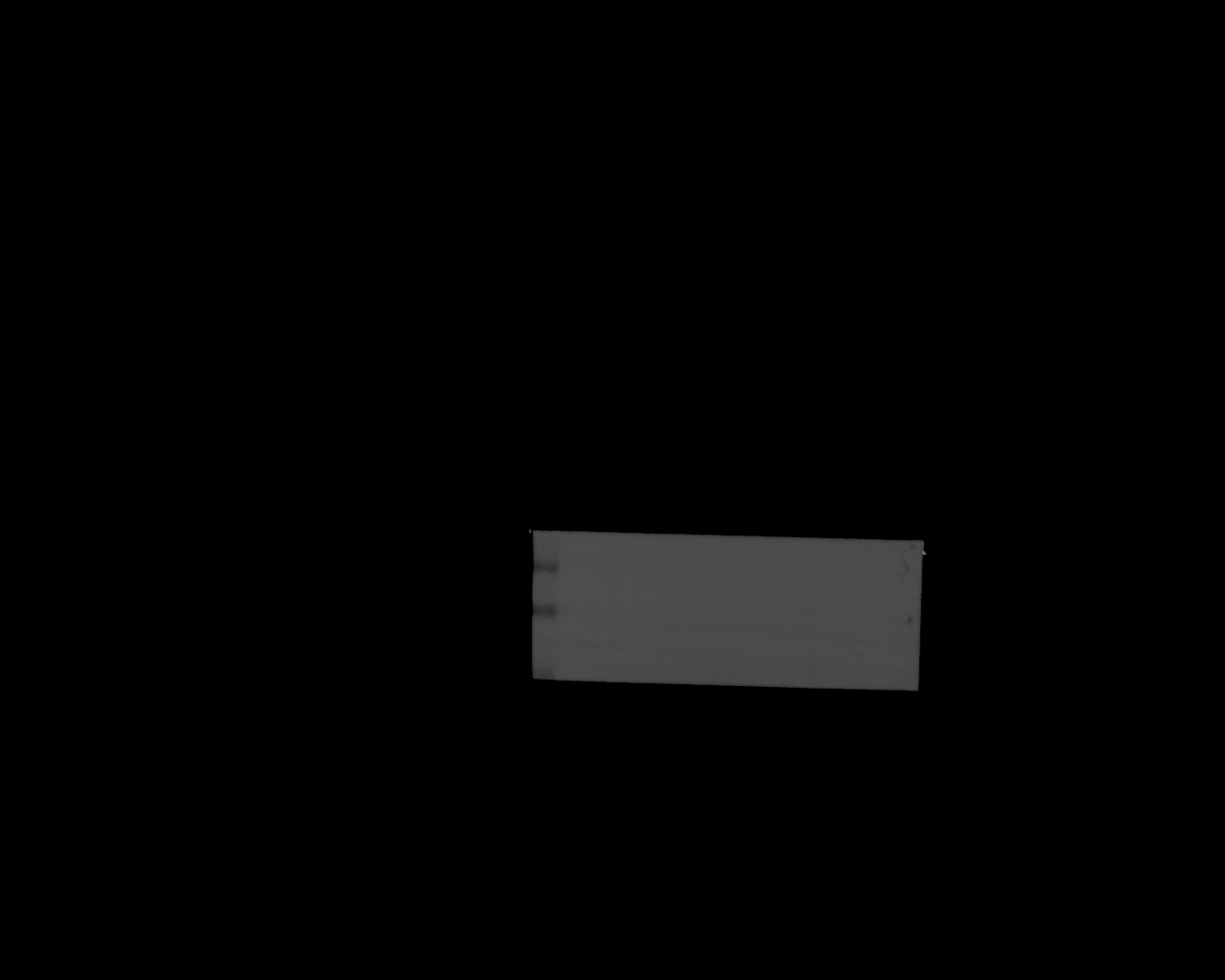

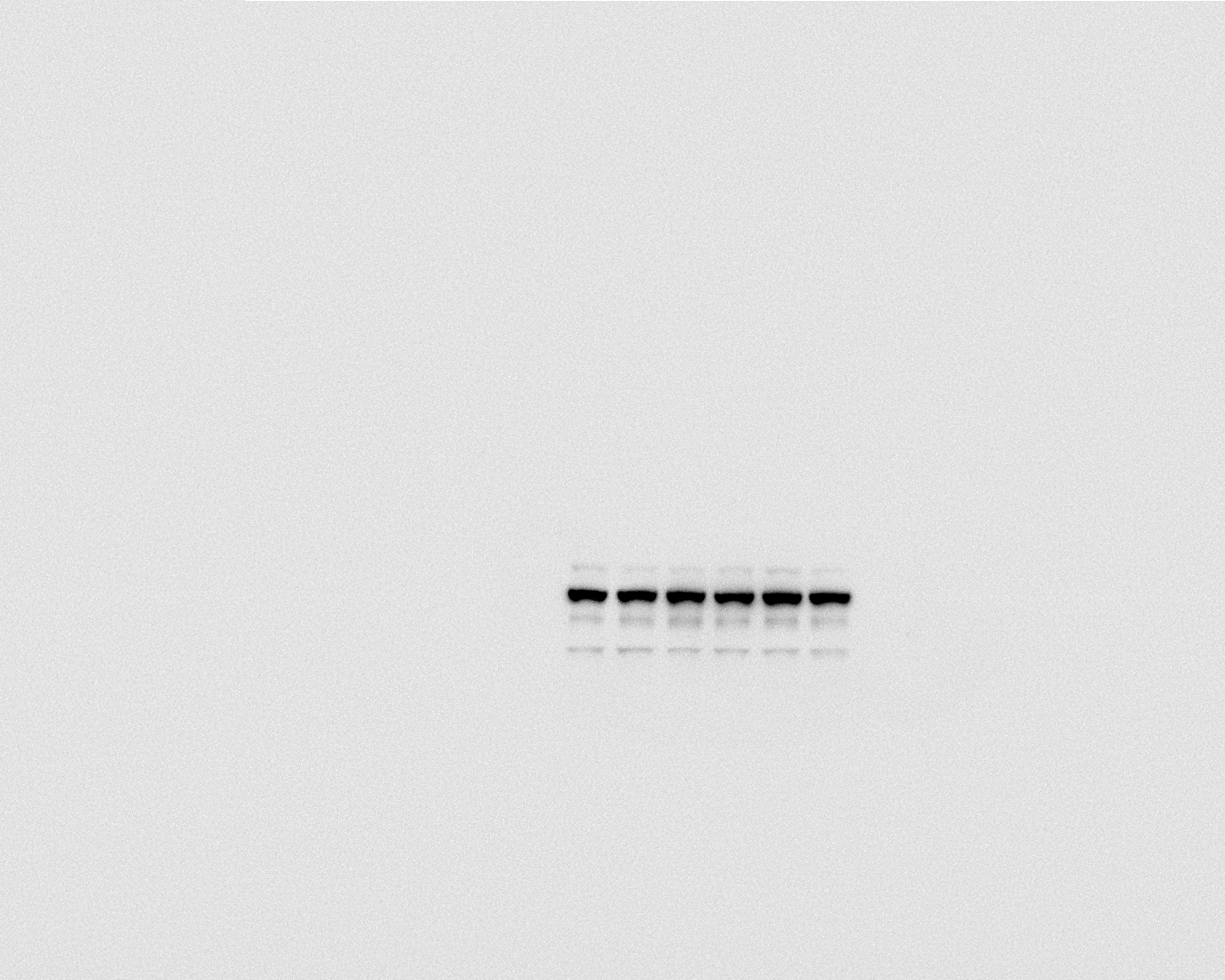

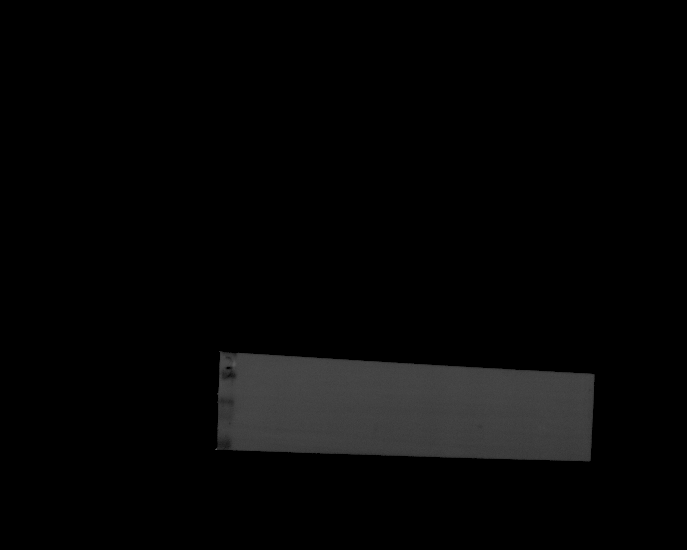

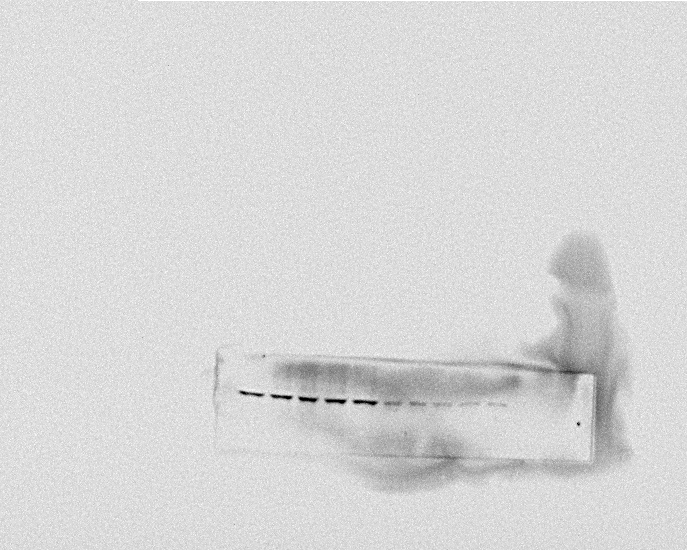

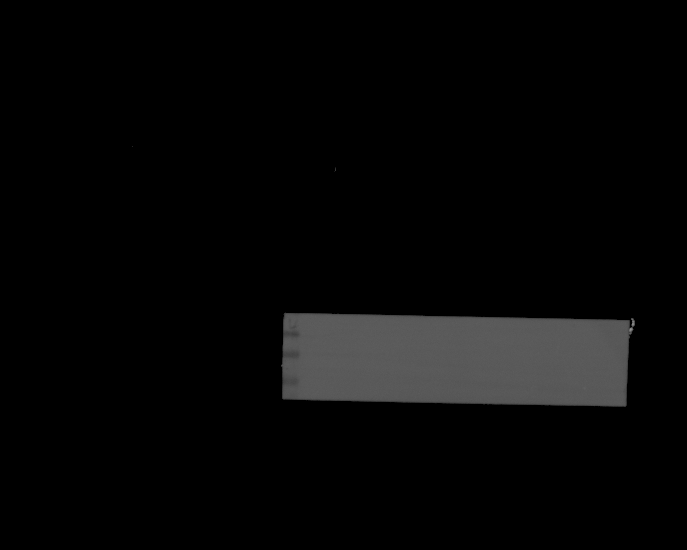

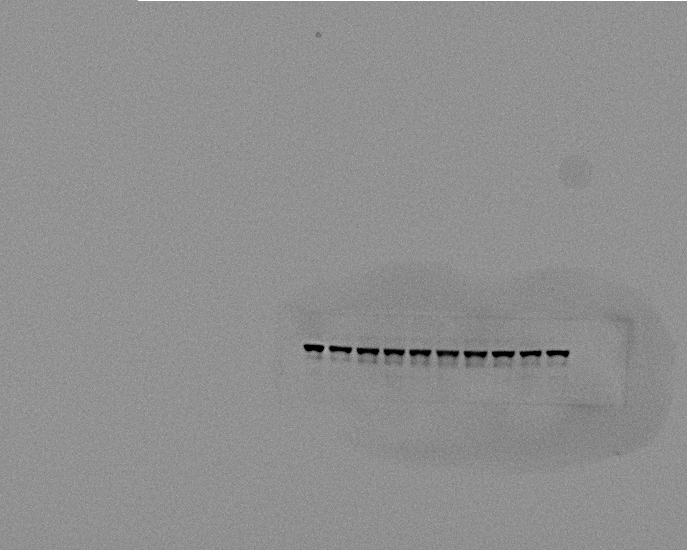

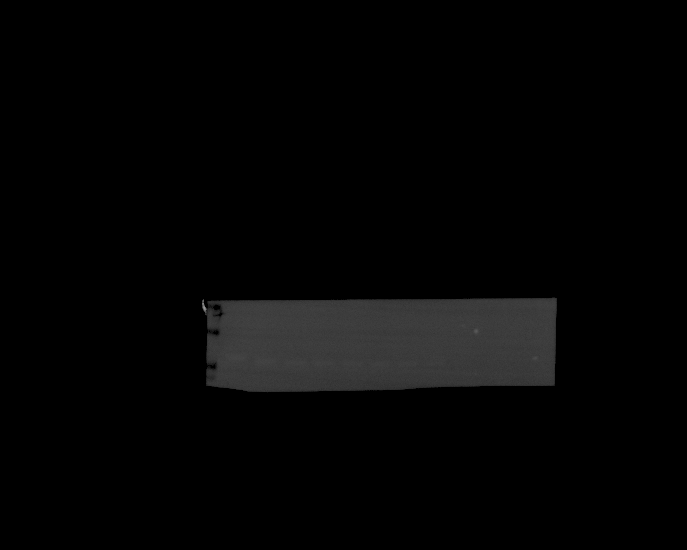

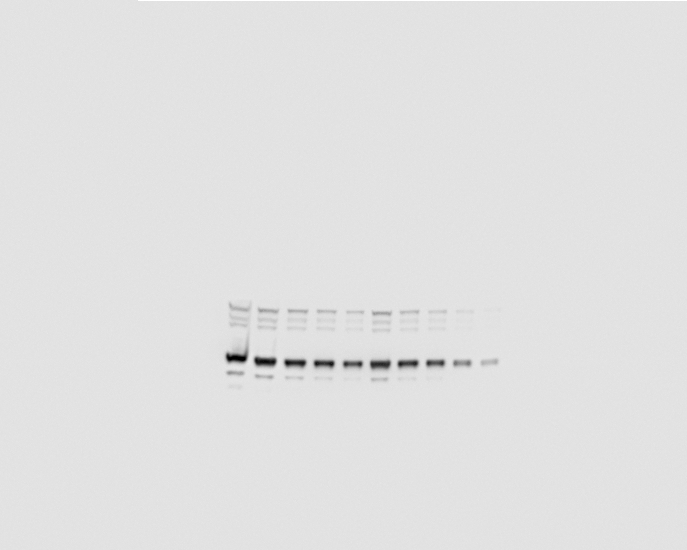


42 kDa

30 kDa

23 kDa

42 kDa

30 kDa

23 kDa

TFPI2

42 kDa

30 kDa

23 kDa

55 kDa

23 kDa

55 kDa

30 kDa

42 kDa

β-actin

30 kDa

55 kDa

42 kDa

30 kDa

55 kDa

42 kDa

**Fig. 6e**

TIRAP

30 kDa

55 kDa

42 kDa

23 kDa

55 kDa

42 kDa

23 kDa

30 kDa

TFPI2

18 kDa

42 kDa

23 kDa

30 kDa

18 kDa

42 kDa

23 kDa

30 kDa

β-actin

55 kDa

30 kDa

42 kDa

55 kDa

30 kDa

42 kDa

**Fig. 6g**

TIRAP


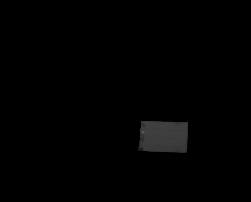

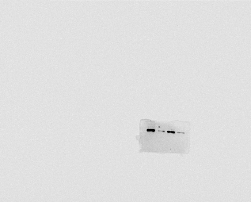

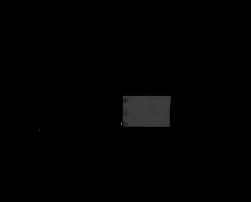

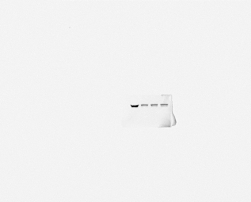


42 kDa

23 kDa

30 kDa

18 kDa

42 kDa

30 kDa

18 kDa

23 kDa

TFPI2

42 kDa

30 kDa

18 kDa

23 kDa

42 kDa

30 kDa

18 kDa

23 kDa

β-actin


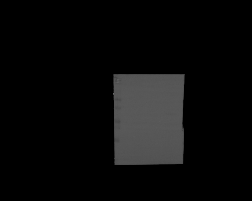

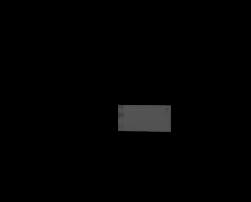

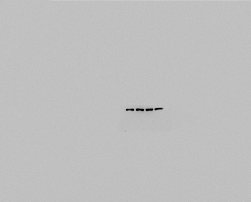

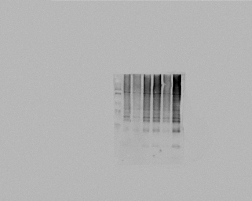

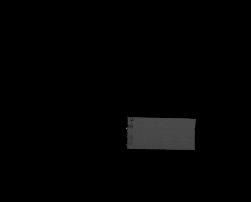

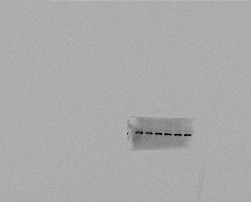


55 kDa

42 kDa

30 kDa

55 kDa

42 kDa

30 kDa

**Fig. 6i**

IP-TIRAP: Ub

55 kDa

42 kDa

30 kDa

23 kDa

18 kDa

200 kDa

110 kDa

75 kDa

140 kDa

42 kDa

30 kDa

23 kDa

18 kDa

200 kDa

75 kDa

140 kDa

110 kDa

55 kDa

IP-TIRAP: TIRAP

42 kDa

30 kDa

23 kDa

42 kDa

30 kDa

23 kDa

Input: Ub


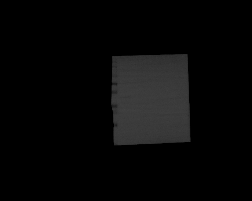

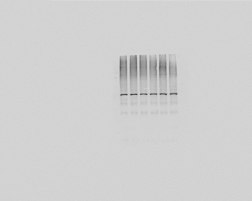


30 kDa

23 kDa

18 kDa

200 kDa

55 kDa

140 kDa

75 kDa

110 kDa

42 kDa

23 kDa

18 kDa

200 kDa

140 kDa

110 kDa

42 kDa

75 kDa

55 kDa

30 kDa

Input: TIRAP


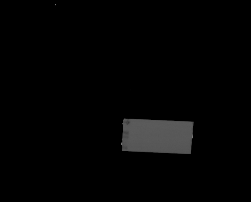

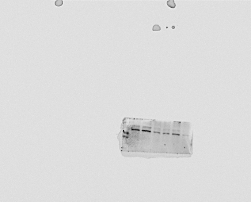


42 kDa

30 kDa

23 kDa

18 kDa

42 kDa

30 kDa

23 kDa

18 kDa

Input: TFPI2


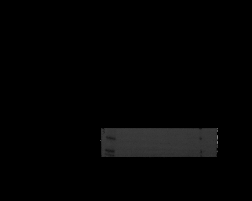

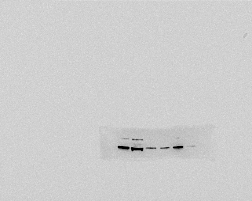


42 kDa

23 kDa

30 kDa

42 kDa

23 kDa

30 kDa

Input: β-actin


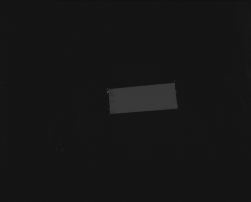

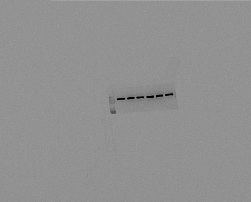


42 kDa

55 kDa

30 kDa

55 kDa

30 kDa

42 kDa

**Fig. 6j**

IP-Myc: HA


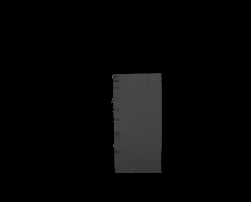

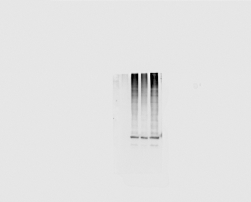


23 kDa

18 kDa

200 kDa

140 kDa

110 kDa

42 kDa

30 kDa

55 kDa

75 kDa

23 kDa

18 kDa

200 kDa

140 kDa

110 kDa

55 kDa

75 kDa

42 kDa

30 kDa

IP-Myc: Myc


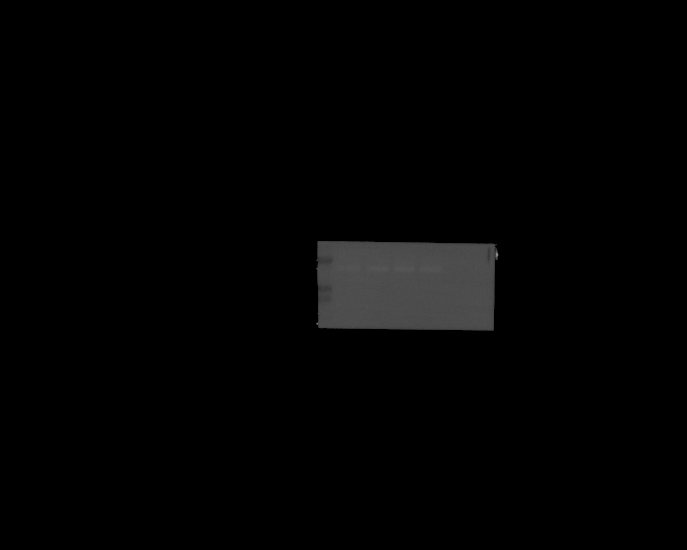

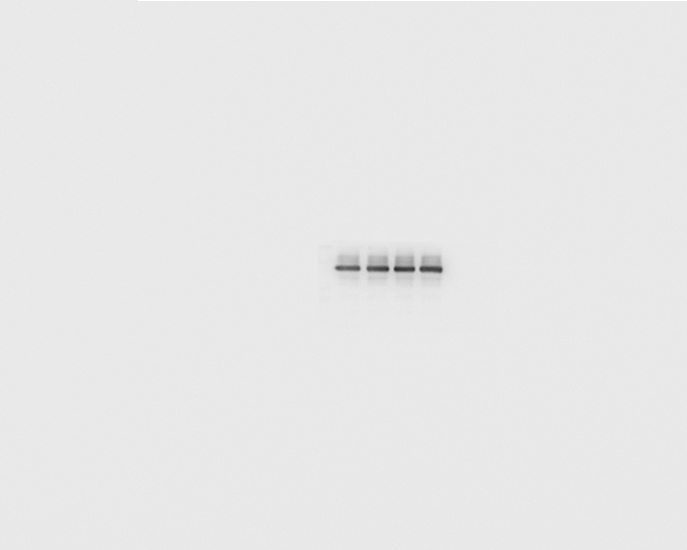


30 kDa

42 kDa

23 kDa

42 kDa

23 kDa

30 kDa

Input: HA


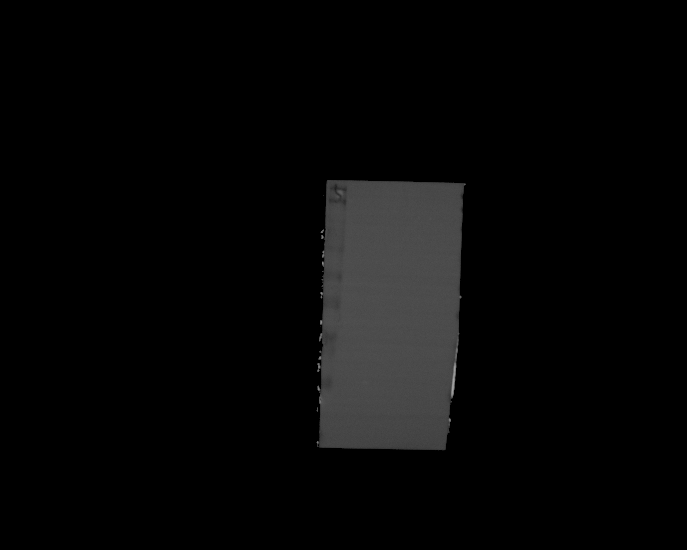

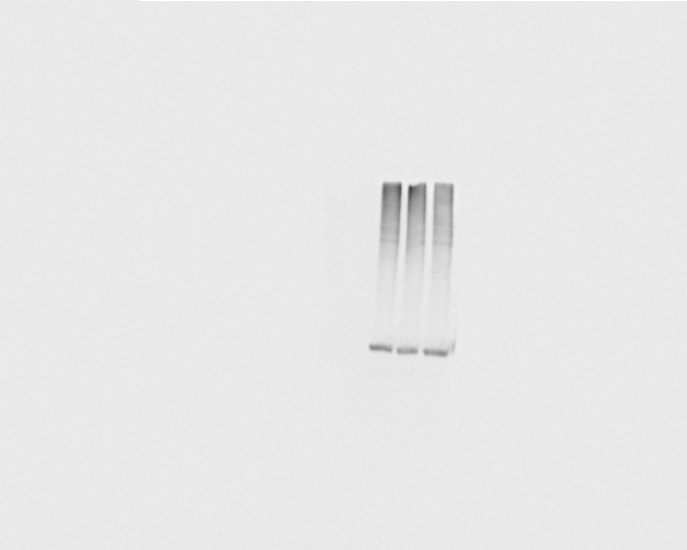


23 kDa

18 kDa

200 kDa

140 kDa

110 kDa

42 kDa

30 kDa

55 kDa

75 kDa

18 kDa

200 kDa

140 kDa

110 kDa

30 kDa

55 kDa

75 kDa

23 kDa

42 kDa

Input: Myc


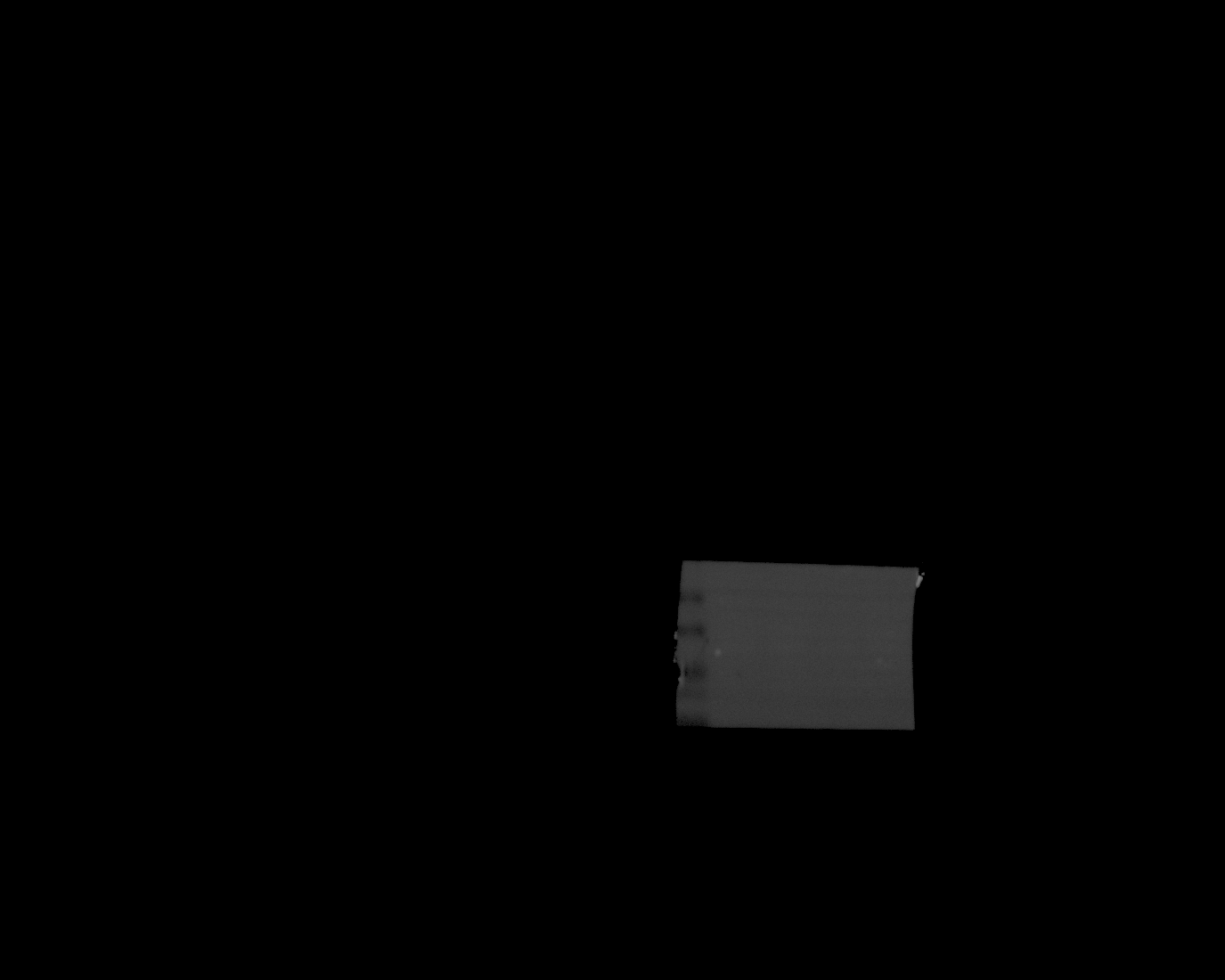


42 kDa

23 kDa

55 kDa

30 kDa

18 kDa

55 kDa

30 kDa

18 kDa

23 kDa

42 kDa

Input: His

55 kDa

30 kDa

23 kDa

42 kDa

75 kDa

23 kDa

42 kDa

75 kDa

55 kDa

30 kDa

Input: TFPI2

23 kDa

30 kDa

42 kDa

23 kDa

30 kDa

42 kDa

**Fig. 6k**

IP-Myc: HA

18 kDa

200 kDa

110 kDa

30 kDa

55 kDa

75 kDa

23 kDa

42 kDa

140 kDa

10 kDa

18 kDa

200 kDa

110 kDa

75 kDa

23 kDa

42 kDa

140 kDa

10 kDa

55 kDa

30 kDa

IP-Myc: Myc

55 kDa

23 kDa

42 kDa

30 kDa

55 kDa

23 kDa

42 kDa

30 kDa

Input: HA

18 kDa

200 kDa

110 kDa

75 kDa

23 kDa

42 kDa

140 kDa

30 kDa

55 kDa

18 kDa

200 kDa

110 kDa

23 kDa

140 kDa

30 kDa

55 kDa

Input: Myc

18 kDa

23 kDa

42 kDa

30 kDa

18 kDa

23 kDa

42 kDa

30 kDa

Input: His

30 kDa

55 kDa

42 kDa

30 kDa

55 kDa

42 kDa

Input: Flag

110 kDa

200 kDa

140 kDa

110 kDa

200 kDa

140 kDa

**Fig. 6l**

Myc-TIRAP

23 kDa

42 kDa

30 kDa

23 kDa

42 kDa

30 kDa

Flag-CLIP1

110 kDa

200 kDa

140 kDa

75 kDa

110 kDa

200 kDa

140 kDa

75 kDa

His-TFPI2

23 kDa

42 kDa

30 kDa

18 kDa

42 kDa

30 kDa

18 kDa

23 kDa

β-actin

55 kDa

42 kDa

30 kDa

55 kDa

42 kDa

30 kDa

**Fig. 7b**

TFPI2

23 kDa

42 kDa

30 kDa

23 kDa

42 kDa

30 kDa

CLIP1

110 kDa

200 kDa

140 kDa

110 kDa

200 kDa

140 kDa

β-actin

30 kDa

55 kDa

42 kDa

30 kDa

55 kDa

42 kDa

**Fig. S7**

IP-Myc: HA

30 kDa

55 kDa

42 kDa

75 kDa

140 kDa

110 kDa

200 kDa

30 kDa

42 kDa

75 kDa

110 kDa

200 kDa

55 kDa

140 kDa

IP-Myc: Myc

30 kDa

42 kDa

23 kDa

42 kDa

23 kDa

30 kDa

Input: HA

30 kDa

42 kDa

110 kDa

200 kDa

140 kDa

55 kDa

75 kDa

30 kDa

42 kDa

110 kDa

200 kDa

140 kDa

55 kDa

75 kDa

Input: Myc

30 kDa

42 kDa

23 kDa

42 kDa

23 kDa

Input: Flag

110 kDa

200 kDa

140 kDa

110 kDa

200 kDa

140 kDa
